# Supplementary material for: Global distribution of surface soil organic carbon in urban greenspaces
Source: Nat Commun. 2024 Jan 27;15:806. doi: 10.1038/s41467-024-44887-y (PMC11258340; doi:10.1038/s41467-024-44887-y)
Supplement: Supplementary file 1 — Supplementary Information [file 41467_2024_44887_MOESM1_ESM.pdf]

Supplementary information

Global distribution of surface soil organic carbon in urban greenspaces

Hongbo Guo[1,2], Enzai Du[1,2*], César Terrer[3], Robert B. Jackson[4,5]

[1]State Key Laboratory of Earth Surface Processes and Resource Ecology, Faculty of Geographical Science, Beijing Normal University, Beijing 100875, China.

[2]School of Natural Resources, Faculty of Geographical Science, Beijing Normal University, Beijing 100875, China.

[3]Department of Civil and Environmental Engineering, Massachusetts Institute of Technology, Cambridge, MA 02139-4307, USA.

[4]Department of Earth System Science, Stanford University, Stanford, CA, USA.

[5]Woods Institute for the Environment and Precourt Institute for Energy, Stanford University, Stanford, CA, USA.

[*] Correspondence to Enzai Du, Email: enzaidu@bnu.edu.cn, Tel: +86-10-58808085

This file includes:

Supplementary Figures 1 to 14, Supplementary Tables 1 to 5, and Supplementary References.

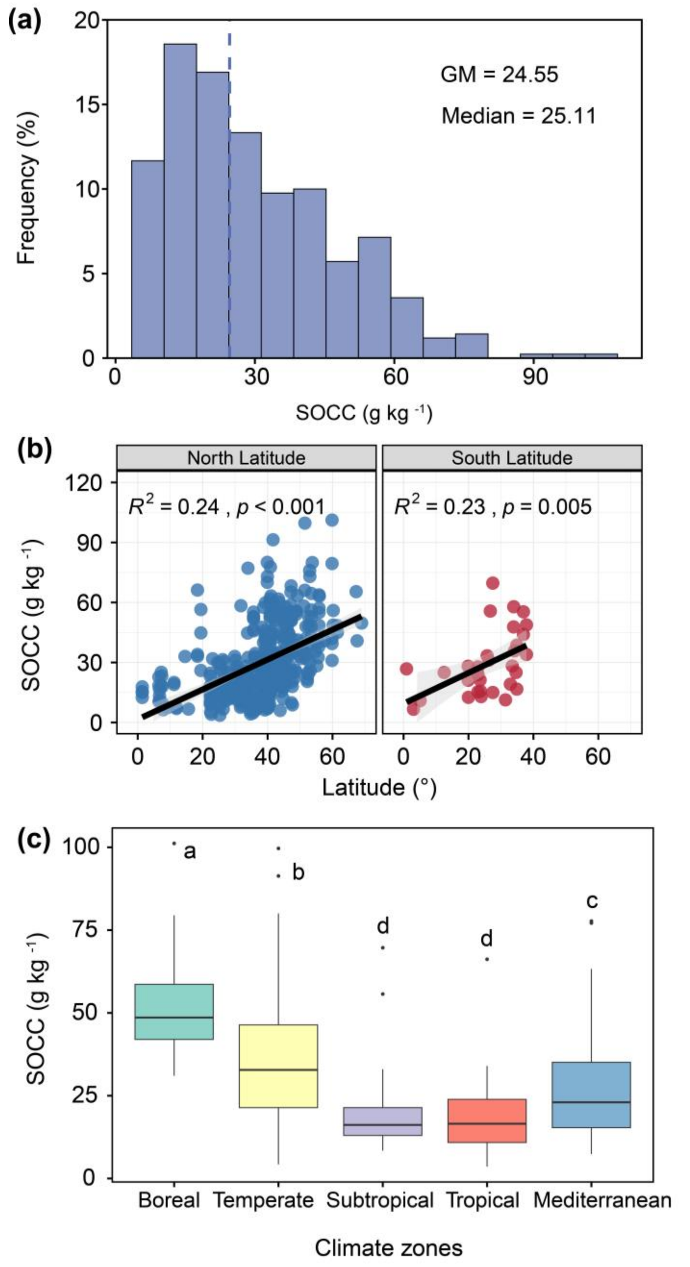

Supplementary Fig. 1. Spatial variation of observed surface SOC concentration (SOCC, g kg^{-1}) (0-20 cm) across global urban greenspaces in SOC-U database. a, The frequency distribution of observed SOCC. **b,** Changes in observed SOCC with latitude for the northern and southern hemisphere. The shaded area represents the 95% confidence interval. **c,** Boxplots showing SOCC in urban greenspaces for different climate zones. Upper and lower bars represent 95th and 5th percentiles. Different letters indicate significant differences for the SOCC among climate zones (LSD test, $P < 0.05$). Source data are provided as a Source Data file.

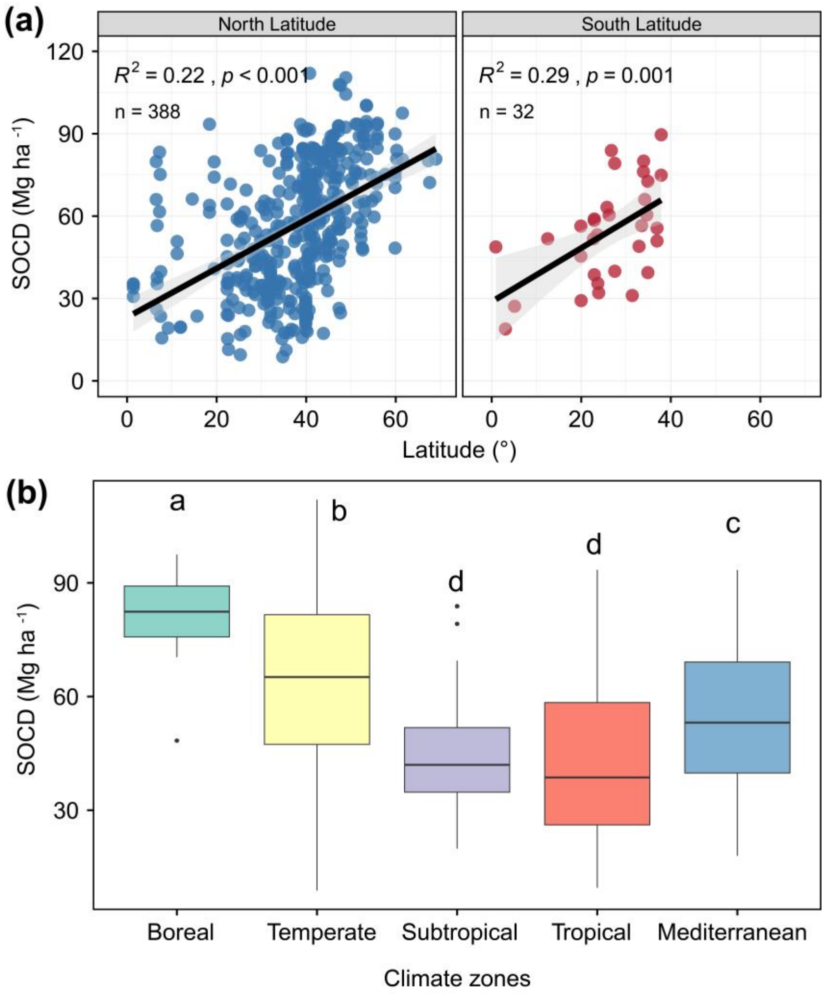

Supplementary Fig. 2. Spatial variation of observed surface SOC density (SOCD, Mg C ha^{-1}) (0-20 cm) across global urban greenspaces in SOC-U database. a, Changes in observed SOCD with latitude for the northern and southern hemisphere. The shaded area represents the 95% confidence interval. **b,** Boxplots showing SOCD in urban greenspaces for different climate zones. Upper and lower bars represent 95th and 5th percentiles. Different letters indicate significant differences for the SOCD among climate zones (LSD test, $P < 0.05$). Source data are provided as a Source Data file.

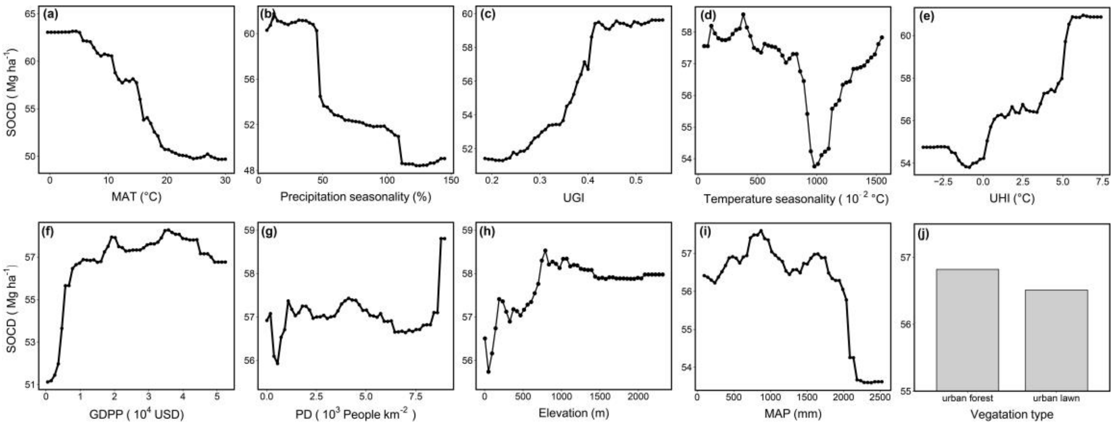

Supplementary Fig. 3. Partial dependence plots indicating the dependence of soil organic carbon density (SOCD) on ten predictors (MAT, MAP, temperature seasonality, precipitation seasonality, UGI, UHI, GDPP, PD, elevation, and vegetation type). Abbreviations: UGI, urban greenness index; MAT, mean annual air temperature; MAP, mean annual precipitation; GDPP, GDP per capita; PD, population density; UHI, urban heat island index. Source data are provided as a Source Data file.

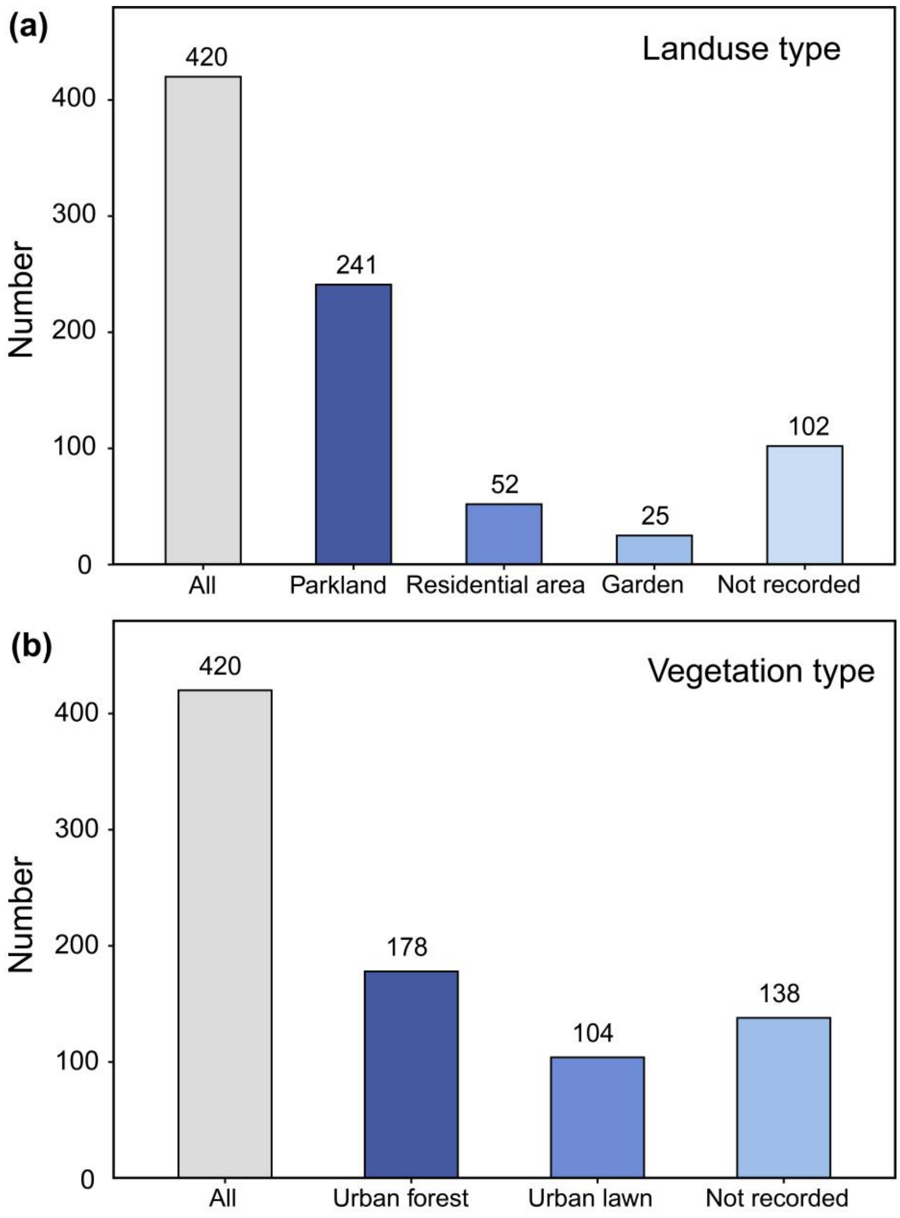

Supplementary Fig. 4. Land use type (a) and vegetation type (b) for urban greenspaces in SOC-U database. The numbers above the bar indicate the sample size. Source data are provided as a Source Data file.

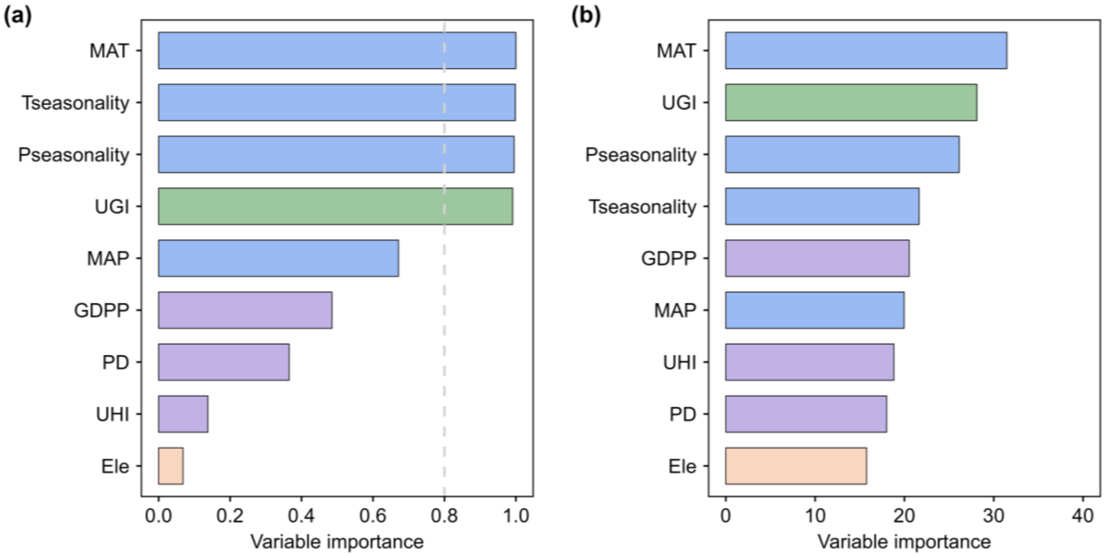

Supplementary Fig. 5. Relative importance of the potential predictors for SOC density (SOCD) based on linear model analysis (a) and random forest analysis (b). All data of SOC-U were used for the analysis (n=420). The variable importance shown in **a** is based on the sum of the Akaike weights derived from model selection using corrected Akaike information criterion. The cut-off is set at 0.8 (dashed line) to differentiate among the important predictors. The importance shown in **b** is based on Mean Decrease Gini of random forest models. Abbreviations: MAT, mean annual air temperature; MAP, mean annual precipitation; Tseasonality, temperature seasonality, Pseasonality, precipitation seasonality; UGI, urban greenness index; GDPP, GDP per capita; PD, population density; UHI, urban heat island index; Ele, city elevation. Source data are provided as a Source Data file.

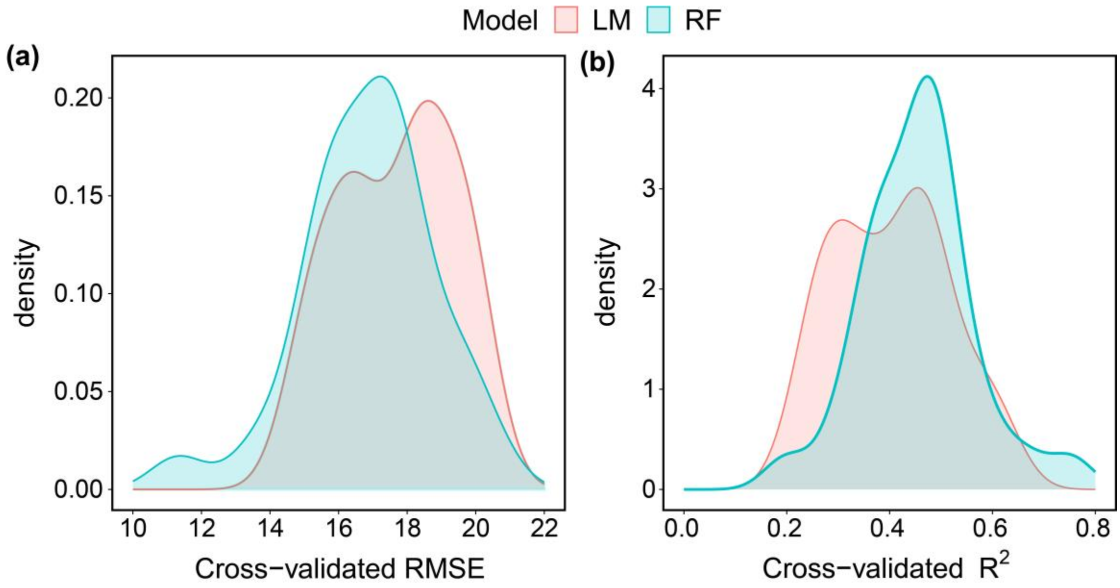

Supplementary Fig. 6. Density plot comparing cross-validated RMSE (a) and cross-validated R^2 (b) for the linear model (LM) and the random-forest model (RF). Source data are provided as a Source Data file.

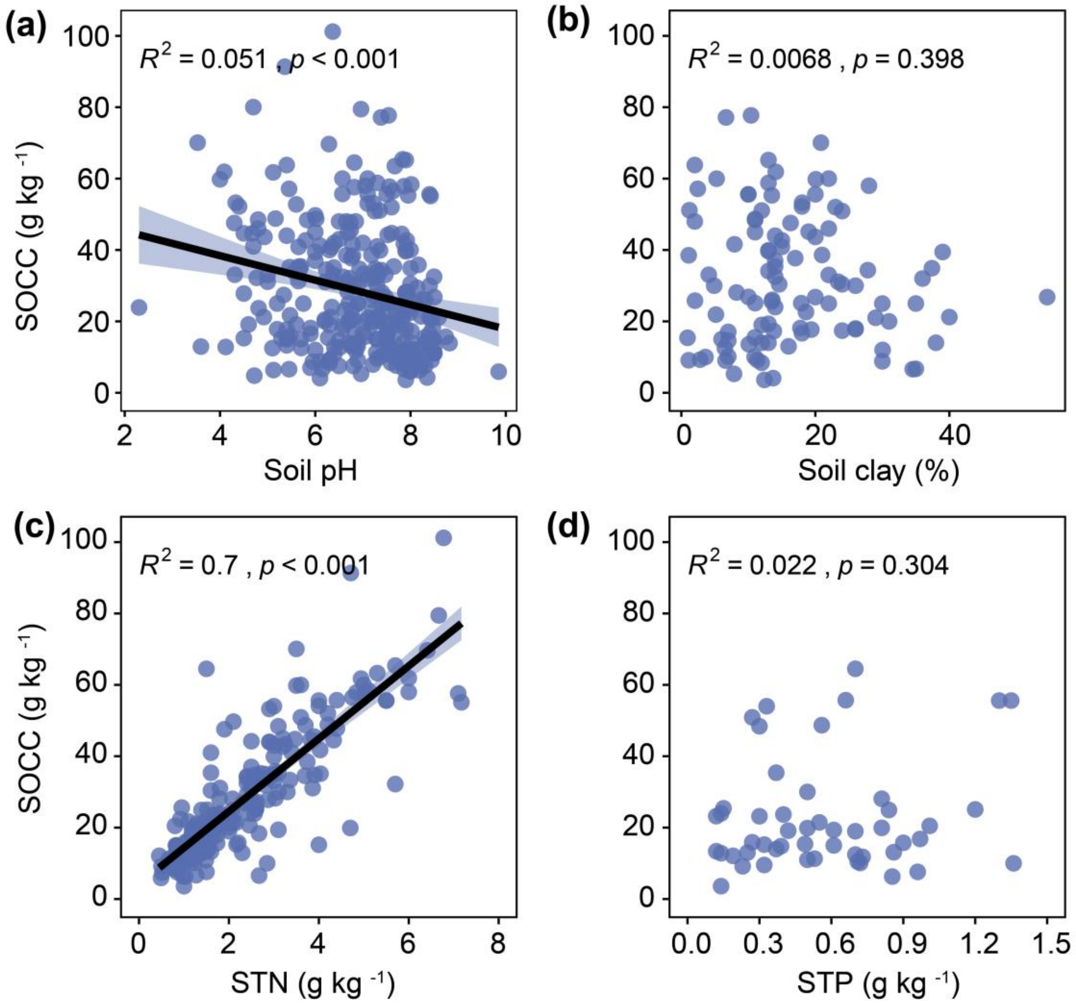

Supplementary Fig. 7. Relationships between surface SOC concentration (SOCC, g kg^{-1}) (0-20 cm) and soil pH (a), soil clay fraction (%) (b), soil total nitrogen concentration (STN, g kg^{-1}) (c), soil total phosphorus concentration (STP, g kg^{-1}) (d) in urban greenspaces. The shaded area represents the 95% confidence interval. Source data are provided as a Source Data file.

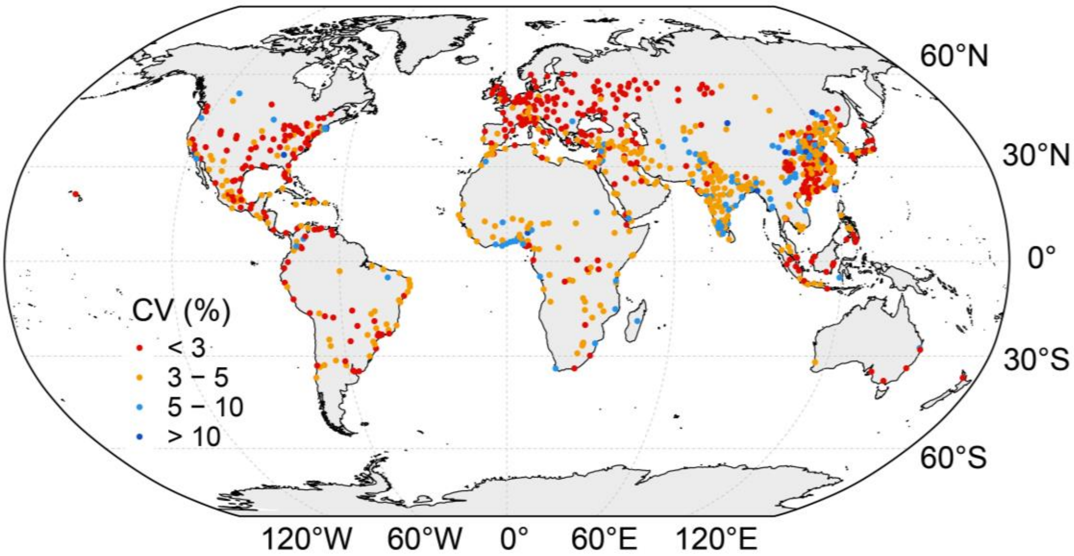

Supplementary Fig. 8. Coefficients of variance (CV, %) of predicted surface soil organic carbon density (SOCD) (0-20 cm) in global urban greenspaces. The world map was created using R 4.0.5 (see more details in Methods section). Source data are provided as a Source Data file.

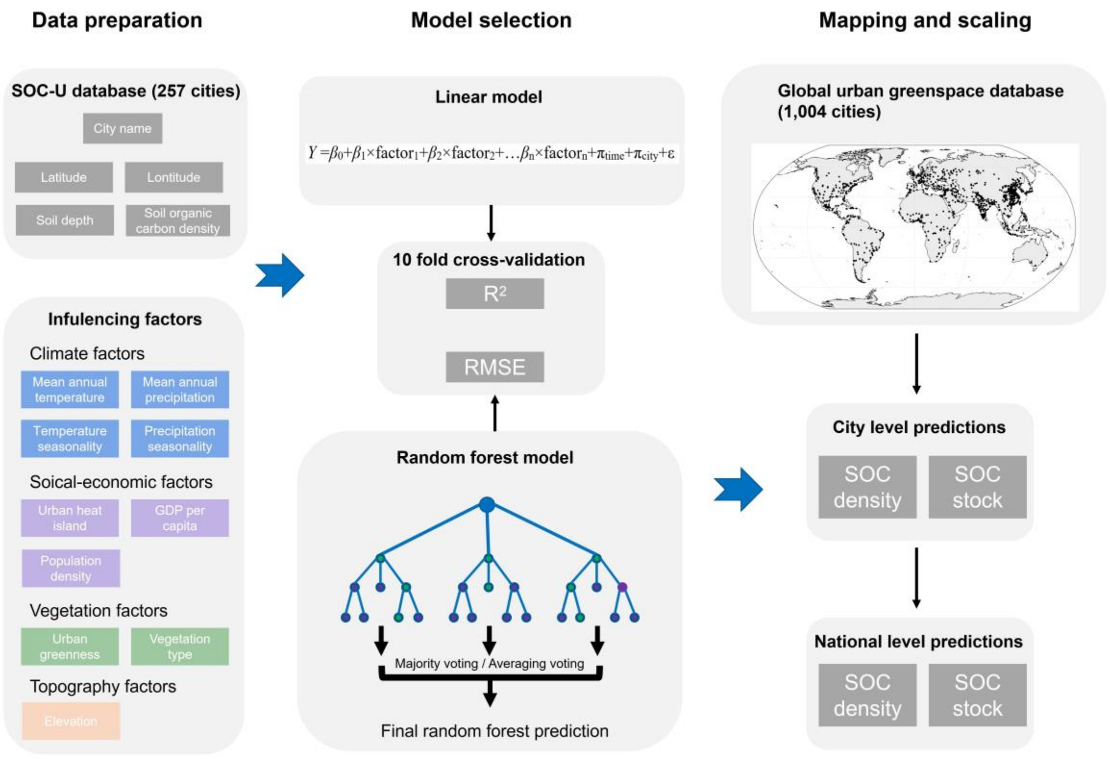

Supplementary Fig. 9. **A conceptual framework for data collection and analysis.** The world map was created using R 4.0.5 (see more details in Methods section). The conceptual figure of the random forest model was created in Microsoft PowerPoint 2019. Source data are provided as a Source Data file.

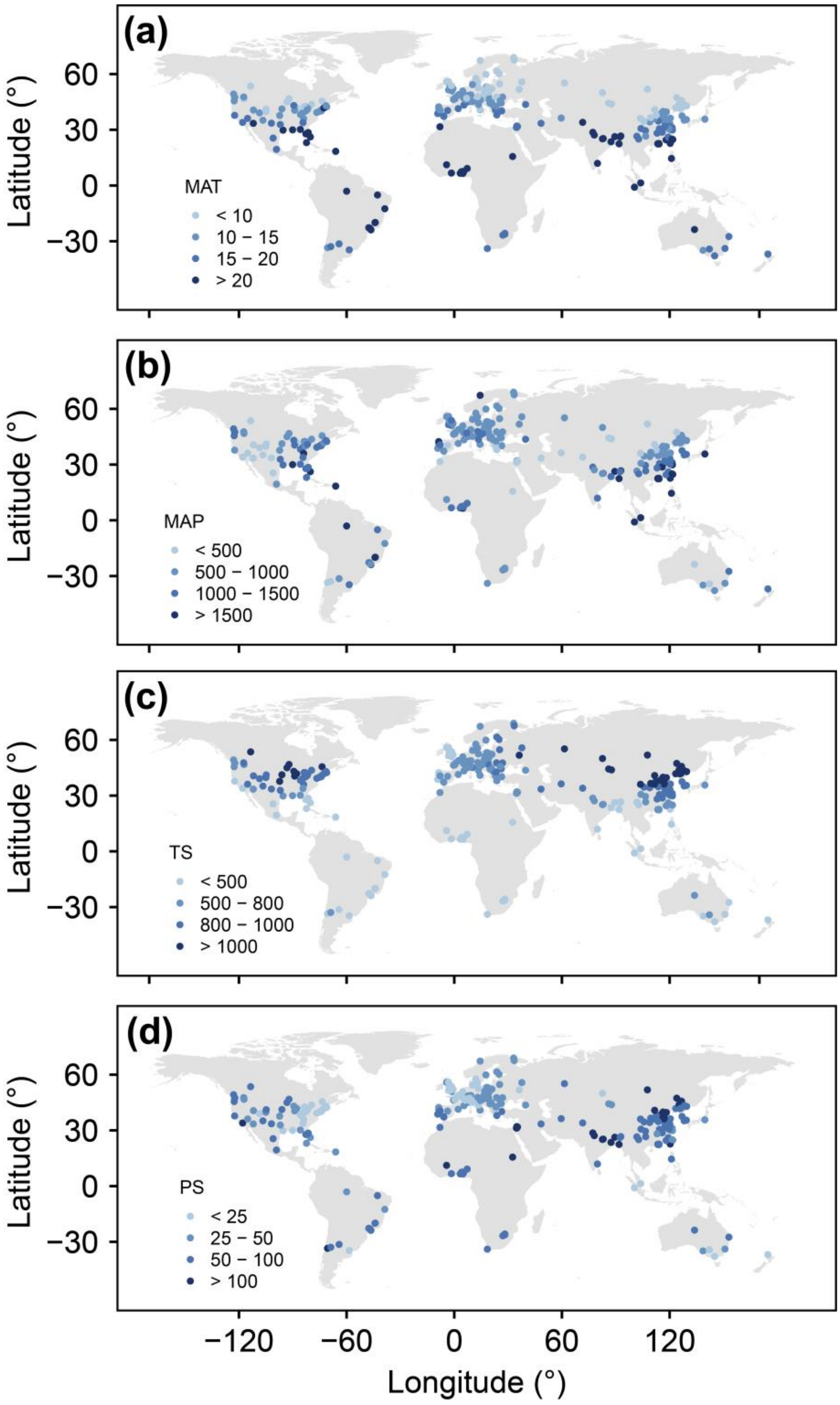

Supplementary Fig. 10. Spatial distribution of climatic variables for sampling cities in the SOC-U database. a-d, mean annual temperature (MAT, ℃) **(a)**, mean annual precipitation (MAP, mm) **(b)**, temperature seasonality (TS, 10^{-2} ℃) **(c)**, and precipitation seasonality (PS, %) **(d)**. The maps were created using R 4.0.5 (see more details in Methods section). Source data are provided as a Source Data file.

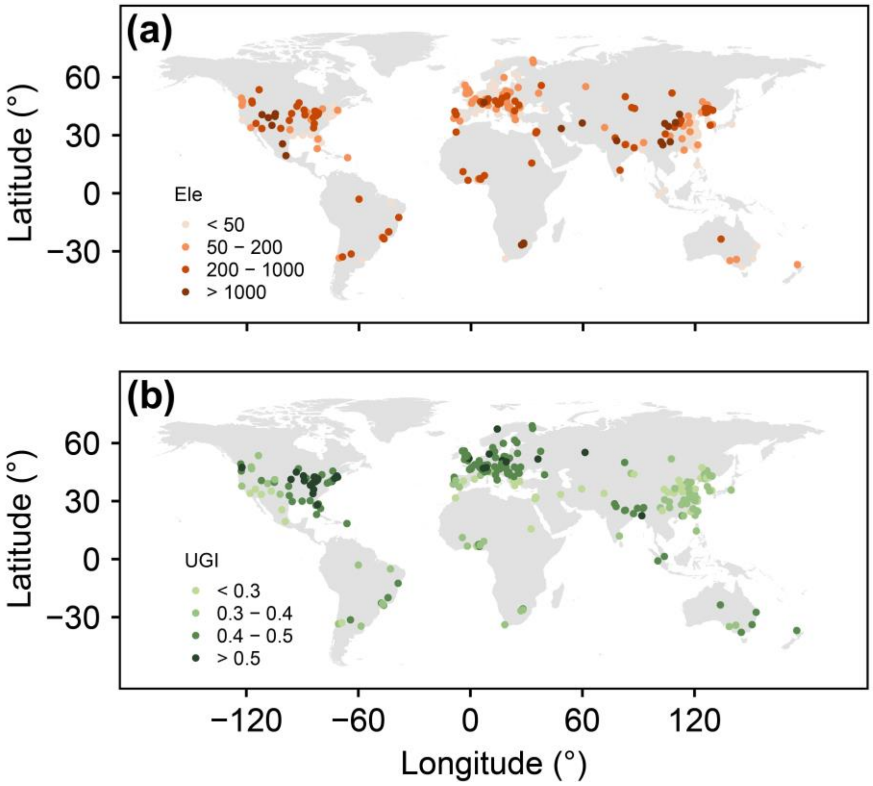

Supplementary Fig. 11. Spatial distribution of topographical and vegetational variables for sampling cities in the SOC-U database. a-b, elevation (Ele, m) **(a)**, and urban greenness index (UGI) **(b)**. The maps were created using R 4.0.5 (see more details in Methods section). Source data are provided as a Source Data file.

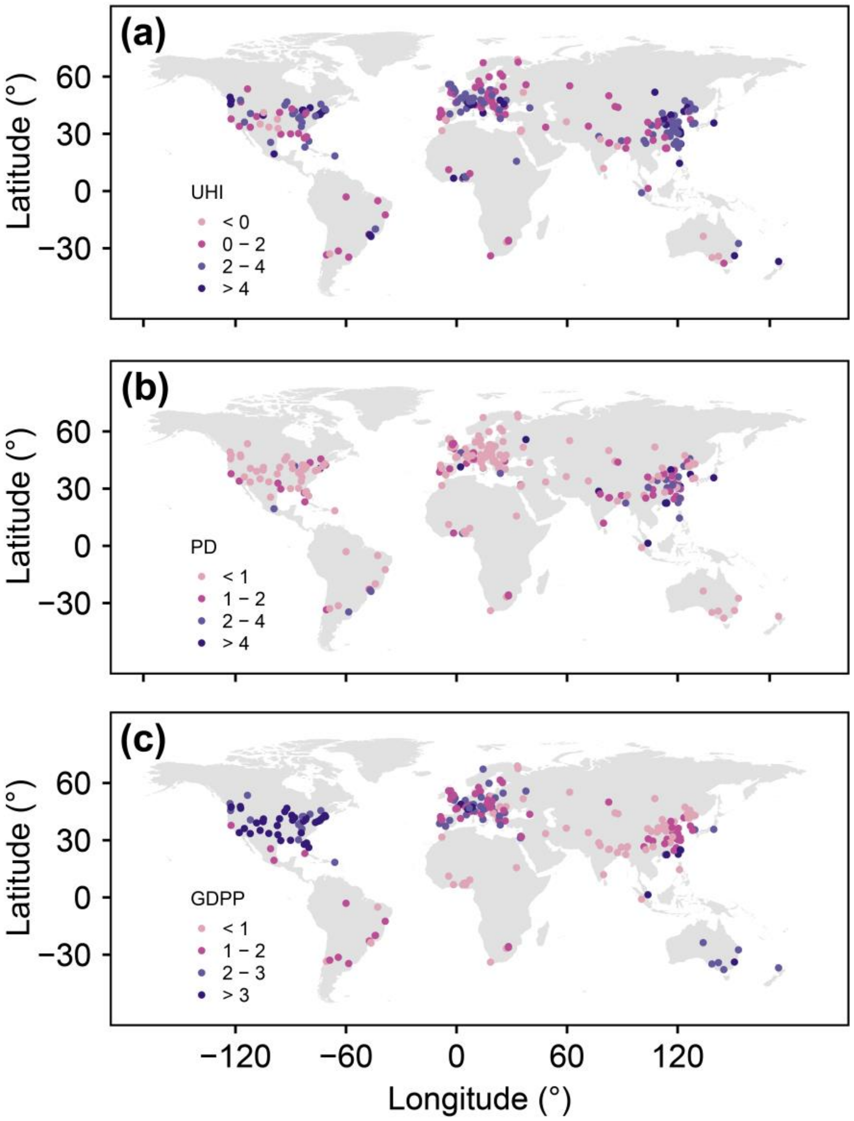

Supplementary Fig. 12. Spatial distribution of social-economic variables for sampling cities in the SOC-U database. a-c, urban heat island index (UHI, ℃) **(a)**, urban population density (PD, 10^3 people km^{-2}) **(b)**, and GDP per capita (GDPP, 10^4 USD) **(c)**. The maps were created using R 4.0.5 (see more details in Methods section). Source data are provided as a Source Data file.

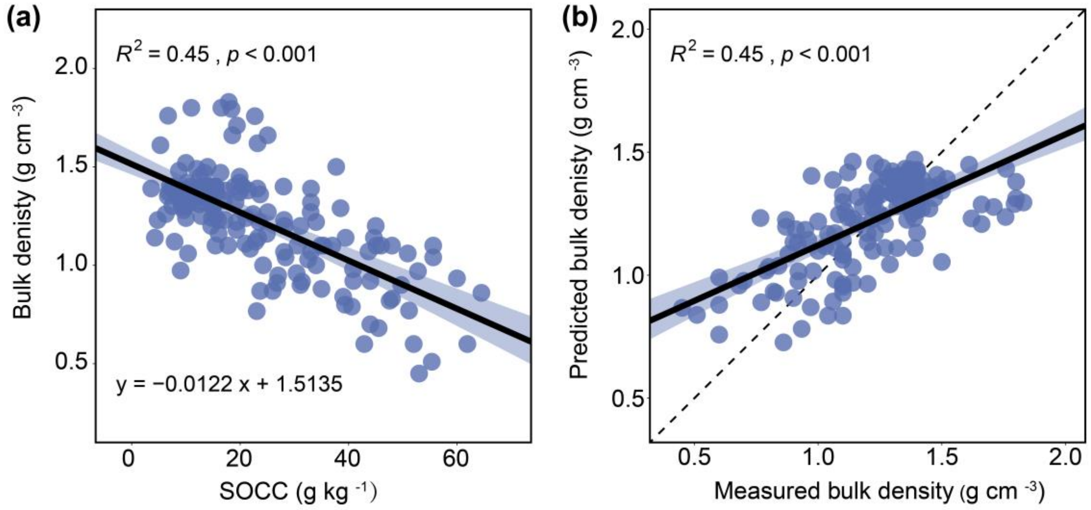

Supplementary Fig. 13. Relationships between observed surface SOC concentration (SOCC, g kg^{-1}) (0-20 cm) and soil bulk density (g cm^{-3}) in urban greenspaces (a) and predicted versus observed values of soil bulk density (b). The shaded area represents the 95% confidence interval. The 1:1 line is plotted in **b**. Source data are provided as a Source Data file.

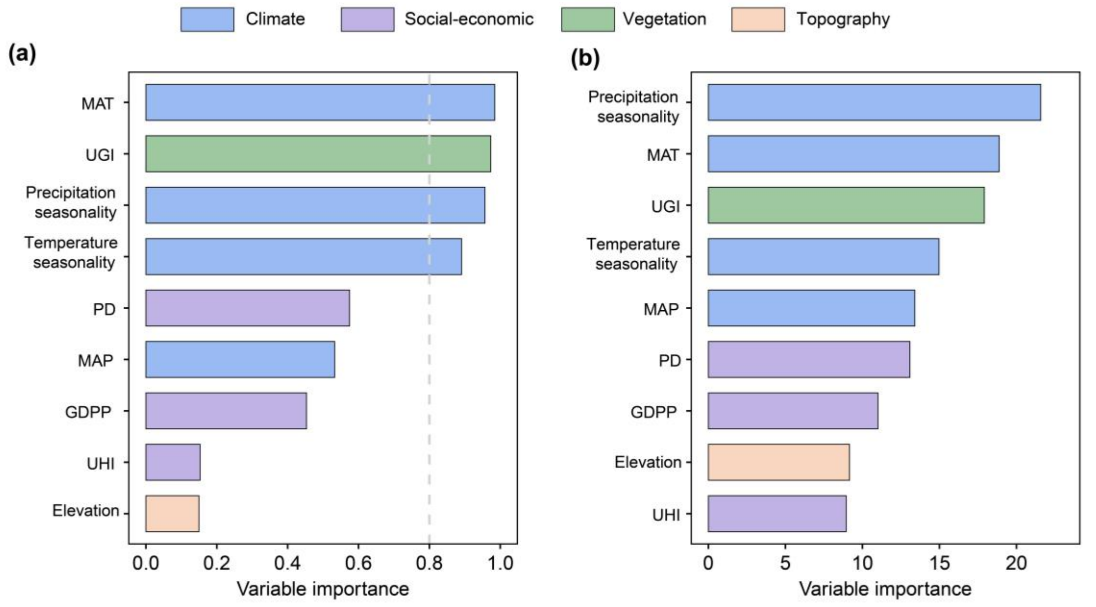

Supplementary Fig. 14. Relative importance of the potential predictors for surface SOC density (SOCD) based on linear model analysis (a) and random forest analysis (b). Only data with reported information on soil bulk density were used for the analysis (n=155). The variable importance shown in **a** is based on the sum of the Akaike weights derived from model selection using corrected Akaike information criterion. The cut-off is set at 0.8 (dashed line) to differentiate among the important predictors. The importance shown in **b** is based on Mean Decrease Gini of random forest models. Abbreviations: UGI, urban greenness index, MAT, mean annual air temperature; MAP, mean annual precipitation; GDPP, GDP per capita; PD, population density, UHI, urban heat island index. Source data are provided as a Source Data file.

Supplementary Table 1. Descriptions of the data sets on potential predictors used for model selection.

ID	Variable	Unit	Type	Source	Reference
1	MAT	°C	Climate	References for the data	/
2	MAP	mm	Climate	References for the data	/
3	Temperature seasonality	10^{-2} °C	Climate	WorldClim database	1
4	Precipitation seasonality	%	Climate	WorldClim database	1
5	Elevation	m	Topography	Global Human Settlement Layer (GHSL) Data Package, 2015	2
6	Vegetation type	/	Vegetation	References for the data	/
7	Urban greenness index	/	Vegetation	Global Human Settlement Layer (GHSL) Data Package, 2015	2, 3
8	Urban heat island index	°C	Socioeconomic	Global Urban Heat Island (UHI) Data Set, 2013	4
9	GDP per capita	USD	Socioeconomic	Global Human Settlement Layer (GHSL) Data Package, 2015	2, 5
10	Population density	People / km^2	Socioeconomic	Gridded Population of the World Version 4 (GPW v4), 2015	6

Supplementary Table 2. Descriptions of the data sets on important predictors used for global prediction and upscaling.

ID	Variable	Unit	Description	Reference
1	MAT	°C	Global Human Settlement Layer (GHSL) Data Package, 2015	2
2	MAP	mm	Global Human Settlement Layer (GHSL) Data Package, 2015	2
3	Elevation	m	Global Human Settlement Layer (GHSL) Data Package, 2015	2
4	Temperature seasonality	10^{-2} °C	WorldClim2 database	1
5	Precipitation seasonality	%	WorldClim2 database	1
6	Urban greenness index	/	Global Human Settlement Layer (GHSL) Data Package, 2015	2, 3
7	Urban heat island index	°C	Global Urban Heat Island (UHI) Data Set, 2013	4
8	GDP per capita	USD	Global Human Settlement Layer (GHSL) Data Package, 2015	2, 5
9	Population density	People / km^2	Gridded Population of the World Version 4 (GPW v4), 2015	6
10	Urban greenspace coverage	%	Global urban green spaces in 1039 cities, 2015	7
11	Urban built-up area	km^2	Global Human Settlement Layer (GHSL) Data Package, 2015	2

Supplementary Table 3. Comparison of model performance among two different models, based on ten-fold cross validation. R^2 and root mean square error (RMSE) were used to evaluate the performance of the models.

**Model**	**SOCD**	
	**R^2**	**RMSE**
Linear regression	0.40	17.64
Random forest	0.46	16.85

Supplementary Table 4. Significance of random factors.

Model:

SOCD20 ~ UGI + MAT + T_seasonality + P_seasonality + (1 | City) + (1 | Year)

	npar	logLik	AIC	LRT	Df	Pr(>Chisq)	
<none>	8	-1785.9	3587.9				
(1 | City)	7	-1802.7	3619.5	33.594	1	6.791e-09	***
(1 | Year)	7	-1785.9	3585.9	0.000	1	1	

Signif. codes: 0 ‘***’ 0.001 ‘**’ 0.01 ‘*’ 0.05 ‘.’

Supplementary Table 5. The global database of soil organic carbon (SOC) concentration and density of greenspaces in urban built-up areas (SOC-U). Abbreviations: Lat, latitude (°); Lon, longitude (°); Depth, soil sampling depth (cm); Ele, city elevation (m); MAT, mean annual air temperature (°C); MAP, mean annual precipitation (mm); TS, temperature seasonality (10^{-2} °C); PS, precipitation seasonality (%); PD, population density (People km^{-2}); GDPP, GDP per capita (USD); UGI, urban greenness index; UHI, urban heat island index (°C); SOCC, soil organic carbon concentration (g kg^{-1}); SOCD, soil organic carbon density (Mg ha^{-1}) ; SOCD20, soil organic carbon density (0-20 cm) (Mg ha^{-1}). Source data are provided as a Source Data file.

ID	City	Lat	Lon	Depth	Vegetation	Ele	MAT	MAP	TS	PS	PD	GDPP	UGI	UHI	SOCC	SOCD	SOCD20	Reference
1	Adelaide	-35	139	10	Urban forest	53	12	551	419	43	427	25729	0.34	-0.85	38.60	40.24	72.66	8
2	Adelaide	-35	139	10	Urban forest	53	12	551	419	43	427	25729	0.34	-0.85	16.67	21.84	39.43	9
3	Athens	38	24	10		155	19	365	681	65	2625	20302	0.27	3.67	26.05	31.15	56.24	10
4	Athens	38	24	20	Urban forest	155	19	365	681	65	2625	20302	0.27	3.67	23.00	56.71	56.71	11
5	Auckland	-37	175	10	Urban forest	53	18	1200	322	20	895	22786	0.47	4.18	55.33	28.22	50.95	12
6	Auckland	-37	175	10	Urban forest	53	18	1200	322	20	895	22786	0.47	4.18	44.00	30.80	55.61	13
7	Aveiro	40	-8	20		16	18	1000	438	51	114	12720	0.38	1.45	13.50	36.42	36.42	14
8	Baltimore	39	-77	10	Urban forest	10	14	1080	897	11	939	32721	0.47	2.86	59.80	46.88	84.64	15
9	Baltimore	39	-77	10	Urban lawn	10	14	1080	897	11	939	32721	0.47	2.86	37.00	40.70	73.48	16
10	Baltimore	39	-77	10	Urban forest	10	14	1080	897	11	939	32721	0.47	2.86	35.38	38.92	70.27	17
11	Baltimore	39	-77	10	Urban lawn	10	14	1080	897	11	939	32721	0.47	2.86	24.00	29.30	52.89	18
12	Beijing	40	116	20	Urban forest	51	12	640	1098	133	6609	16241	0.33	4.90	6.80	18.63	18.63	19
13	Beijing	40	116	20	Urban forest	51	12	640	1098	133	6609	16241	0.33	4.90	8.21	22.33	22.33	20
14	Beijing	40	116	15		51	12	640	1098	133	6609	16241	0.33	4.90	9.10	18.75	23.76	21
15	Beijing	40	116	20		51	12	640	1098	133	6609	16241	0.33	4.90	9.71	27.00	27.00	22
16	Beijing	40	116	20	Urban forest	51	12	640	1098	133	6609	16241	0.33	4.90	10.00	30.40	30.40	23

ID	City	Lat	Lon	Depth	Vegetation	Ele	MAT	MAP	TS	PS	PD	GDPP	UGI	UHI	SOCC	SOCD	SOCD20	Reference
17	Belgrade	45	20	20		117	12	700	784	26	560	16084	0.45	0.85	12.00	32.81	32.81	24
18	Belgrade	45	20	10	Urban forest	117	12	700	784	26	560	16084	0.45	0.85	55.60	46.44	83.84	25
19	Berlin	53	13	20	Urban forest	35	9	580	698	18	878	23559	0.49	2.02	24.23	48.46	48.46	26
20	Bloomington	40	-89	15	Urban forest	252	12	1207	1040	28	43	31723	0.55	2.28	39.33	47.20	59.82	27
21	Bobo-Dioulasso	11	-4	20		460	28	900	181	106	292	1191	0.33	1.76	20.00	50.78	50.78	28
22	Boston	42	-71	15	Urban forest	14	14	1050	926	8	1094	43495	0.52	2.77	48.00	59.76	75.74	29
23	Boston	42	-71	15	Urban forest	14	14	1050	926	8	1094	43495	0.52	2.77	51.14	59.07	74.86	30
24	Brasov	46	26	10	Urban forest	774	11	832	795	50	106	14097	0.41	5.11	32.00	35.94	64.89	31
25	Budapest	47	19	10	Urban lawn	100	11	533	781	25	557	27860	0.45	1.97	25.40	30.57	55.20	18
26	Buenos Aires	-35	-59	20	Urban forest	31	17	1146	490	25	2700	18763	0.34	0.61	25.00	60.43	60.43	32
27	Campinas	-23	-47	10	Urban forest	693	21	1462	229	67	1007	17644	0.36	4.44	15.29	21.41	38.65	33
28	Carpi	45	10	15	Urban forest	29	14	657	806	20	246	15696	0.38	0.37	23.30	39.84	50.50	34
29	Changchun	44	125	20	Urban forest	300	5	650	1392	110	1771	8323	0.34	4.36	21.46	59.22	59.22	35
30	Changsha	28	113	15	Urban forest	54	17	1360	869	48	897	10579	0.31	0.42	19.14	37.90	48.04	36
31	Chattogram	22	92	10		20	29	2800	316	106	4018	3932	0.54	0.54	5.29	8.52	15.38	37
32	Chengdu	31	104	20	Urban forest	500	16	945	710	99	3336	13406	0.33	0.67	17.15	42.20	42.20	38
33	Chengdu	31	104	20		500	16	945	710	99	3336	13406	0.33	0.67	17.89	65.48	65.48	39
34	Chengdu	31	104	10	Urban forest	500	16	945	710	99	3336	13406	0.33	0.67	28.09	34.55	62.38	40
35	Chicago	42	-88	10	Urban lawn	179	10	96	1040	27	2070	35379	0.48	3.96	58.00	46.74	84.39	41
36	Chicago	42	-88	10		179	10	96	1040	27	2070	35379	0.48	3.96	60.00	46.89	84.66	41
37	Chongqing	30	107	20		163	18	1200	751	68	1110	12697	0.39	2.39	19.34	45.64	45.64	42
38	Columbus	40	-83	20	Urban forest	235	11	950	943	22	216	30360	0.51	3.38	35.00	61.60	61.60	43
39	Cooch Behar	26	89	20	Urban forest	43	29	2305	409	109	1041	9543	0.47	2.60	6.66	18.00	18.00	44
40	Cordoba	-31	-64	20	Urban forest	500	18	750	469	73	558	15093	0.43	1.04	11.30	31.09	31.09	45
41	Coventry	52	-1	15	Urban lawn	86	10	800	462	11	275	15309	0.47	2.15	25.00	45.32	57.44	46
42	Dongguan	23	113	10	Urban forest	21	22	1796	538	72	3337	6855	0.34	1.98	15.95	19.62	35.42	47

ID	City	Lat	Lon	Depth	Vegetation	Ele	MAT	MAP	TS	PS	PD	GDPP	UGI	UHI	SOCC	SOCD	SOCD20	Reference
43	Franklin	30	-86	10	Urban forest	4	20	1425	616	30	110	27901	0.42	0.23	31.11	28.00	50.55	48
44	Fuzhou	26	119	20	Urban forest	30	20	1348	671	59	1283	12618	0.32	3.15	12.50	35.00	35.00	49
45	Glasgow	56	-4	20		44	9	900	415	30	821	16967	0.47	2.02	48.50	89.41	89.41	14
46	Guangzhou	22	113	20	Urban forest	22	21	1720	539	74	5063	22218	0.43	1.98	12.82	35.64	35.64	50
47	Guangzhou	22	113	20	Urban forest	22	21	1720	539	74	5063	22218	0.43	1.98	17.00	45.22	45.22	51
48	Guangzhou	22	113	20	Urban forest	22	21	1720	539	74	5063	22218	0.43	1.98	23.18	64.44	64.44	52
49	Hangzhou	30	120	20		19	18	1454	869	44	3097	14873	0.32	3.31	12.84	34.84	34.84	53
50	Hangzhou	30	120	15	Urban forest	19	18	1454	869	44	3097	14873	0.32	3.31	16.39	36.13	45.80	54
51	Hefei	32	117	20	Urban forest	120	17	1300	922	53	2085	6347	0.32	2.51	23.68	64.41	64.41	55
52	Helsinki	60	25	10	Urban forest	25	5	682	821	30	718	18877	0.43	1.94	56.00	46.50	83.95	56
53	Montreal	46	-74	20	Urban forest	33	7	897	1149	13	1127	22658	0.41	2.76	44.22	86.14	86.14	57
54	Helsinki	60	25	10	Urban lawn	25	5	682	821	30	718	18877	0.43	1.94	48.70	44.77	80.84	18
55	Hohhot	41	112	20		1050	8	450	1253	108	979	9088	0.27	3.36	14.91	38.76	38.76	58
56	Ibadan	7	4	20		170	27	1270	129	64	952	6226	0.33	4.84	23.20	75.17	75.17	59
57	Jinan	37	117	20	Urban forest	142	14	670	1064	112	1330	11609	0.31	2.77	6.56	18.81	18.81	60
58	Jinju-si	35	128	20		350	12	1300	916	81	235	12932	0.35	2.90	9.02	17.56	17.56	61
59	Kiel	54	10	10	Urban forest	21	12	775	598	18	319	17501	0.51	1.03	41.00	41.55	75.01	62
60	Kumasi	7	-2	15	Urban forest	258	26	1250	107	53	1288	2935	0.35	4.92	11.00	29.70	37.64	63
61	Kumasi	7	-2	15	Urban forest	258	26	1250	107	53	1288	2935	0.35	4.92	16.53	44.63	56.57	64
62	Kursk	52	36	10		196	10	607	1009	25	221	6451	0.50	-0.52	32.10	36.01	65.02	65
63	Lahti	61	24	10	Urban lawn	40	5	636	868	33	90	12500	0.42	1.10	48.40	44.67	80.66	18
64	Lahti	61	24	10	Urban lawn	40	5	636	868	33	90	12500	0.42	1.10	45.00	54.00	97.50	16
65	Lanzhou	36	104	20	Urban forest	1520	10	311	1011	93	1088	8200	0.24	0.79	11.02	29.10	29.10	66
66	Leicester	53	-1	20	Urban forest	51	8	606	479	10	1016	15603	0.43	2.15	53.00	47.70	47.70	67
67	Leuven	51	5	20	Urban forest	10	11	729	554	12	382	27823	0.48	2.05	35.38	76.56	76.56	68
68	Liverpool	53	-3	15		50	9	796	445	20	943	16099	0.43	1.93	48.00	79.20	100.38	69

ID	City	Lat	Lon	Depth	Vegetation	Ele	MAT	MAP	TS	PS	PD	GDPP	UGI	UHI	SOCC	SOCD	SOCD20	Reference
69	Ljubljana	46	15	20		302	10	1452	768	25	161	22088	0.47	3.94	48.00	89.08	89.08	14
70	Louisville	38	-86	10	Urban forest	142	14	1130	898	16	432	28029	0.54	3.63	47.56	39.00	70.42	70
71	Louisville	38	-86	15	Urban forest	142	14	1130	898	16	432	28029	0.54	3.63	31.11	56.00	70.98	71
72	Madison	43	-89	20	Urban forest	267	18	1300	1077	39	191	30554	0.52	1.39	28.09	60.00	60.00	72
73	Manchester	53	-2	10		47	10	600	449	19	1114	31530	0.50	1.93	52.79	51.20	92.45	73
74	Quezon City [Manila]	15	121	10	Urban forest	10	27	1666	108	88	3909	9118	0.35	5.95	33.00	36.66	66.19	74
75	Marrakesh	32	-8	20	Urban forest	454	25	240	576	64	481	4967	0.24	-0.54	7.60	21.28	21.28	75
76	Marrakesh	32	-8	15		454	25	240	576	64	481	4967	0.24	-0.54	11.11	22.96	29.11	76
77	Marseille	43	5	10		12	16	515	576	43	1534	20314	0.39	2.66	54.00	46.15	83.33	77
78	Melbourne	-38	145	20		27	16	647	375	14	964	27030	0.40	1.96	48.80	89.61	89.61	78
79	Milan	46	9	20	Urban forest	100	14	920	755	23	1374	24235	0.43	4.30	22.83	51.14	51.14	79
80	Moscow	56	38	10		200	7	706	983	31	6823	20523	0.47	1.92	37.80	39.78	71.82	80
81	Moscow	56	38	10		200	7	706	983	31	6823	20523	0.47	1.92	40.00	41.02	74.06	81
82	Moscow, Idaho	48	-117	15	Urban forest	790	10	760	824	37	223	31465	0.39	3.79	37.71	84.85	107.55	82
83	Murmansk	69	33	10	Urban forest	150	4	500	797	37	190	9674	0.42	-0.46	49.70	44.73	80.76	83
84	Nanchang	29	116	20	Urban forest	25	18	1700	866	60	1520	6467	0.38	2.81	15.02	41.76	41.76	84
85	Nanchang	29	116	15	Urban forest	25	18	1700	866	60	1520	6467	0.38	2.81	20.49	43.03	54.54	85
86	Nanjing	32	119	20	Urban forest	21	16	1091	930	58	2687	15062	0.31	2.83	8.40	23.18	23.18	86
87	Nanjing	32	119	20		21	16	1091	930	58	2687	15062	0.31	2.83	13.92	38.98	38.98	87
88	Nanjing	32	119	10	Urban forest	21	16	1091	930	58	2687	15062	0.31	2.83	15.52	21.73	39.23	88
89	Nanjing	32	119	10	Urban forest	21	16	1091	930	58	2687	15062	0.31	2.83	17.27	23.83	43.03	89
90	New York	41	-74	10	Urban forest	10	13	1174	893	10	6041	36082	0.49	4.34	61.90	37.14	67.06	90
91	New York	41	-74	20	Urban forest	10	13	1174	893	10	6041	36082	0.49	4.34	59.98	111.99	111.99	91
92	New York	41	-74	10	Urban forest	10	13	1174	893	10	6041	36082	0.49	4.34	53.25	46.00	83.05	15
93	New York	41	-74	20	Urban forest	10	13	1174	893	10	6041	36082	0.49	4.34	52.20	91.53	91.53	92
94	New York	41	-74	10	Urban forest	10	13	1174	893	10	6041	36082	0.49	4.34	42.89	25.73	46.46	93

ID	City	Lat	Lon	Depth	Vegetation	Ele	MAT	MAP	TS	PS	PD	GDPP	UGI	UHI	SOCC	SOCD	SOCD20	Reference
95	New York	41	-74	10	Urban forest	10	13	1174	893	10	6041	36082	0.49	4.34	39.00	32.76	59.15	94
96	Newark	40	-74	10	Urban forest	10	13	1140	861	10	621	28171	0.53	4.34	41.00	41.55	75.01	95
97	Ningbo	30	122	20	Urban forest	4	16	1480	827	41	1830	14155	0.30	4.86	33.00	83.82	83.82	96
98	Obrenovac	44	20	10	Urban forest	117	12	700	764	29	52	4274	0.47	0.64	21.00	26.40	47.67	25
99	Pancevo	45	21	10	Urban forest	78	12	700	783	26	106	4474	0.45	0.85	30.00	34.43	62.15	25
100	Paris	49	2	10	Urban forest	108	11	600	565	11	4248	34274	0.43	2.64	55.60	57.82	104.40	97
101	Paris	49	2	10	Urban lawn	108	11	600	565	11	4248	34274	0.43	2.64	25.10	31.88	57.55	97
102	Paris	49	2	10	Urban forest	108	11	600	565	11	4248	34274	0.43	2.64	55.60	61.16	110.42	98
103	Philadelphia	40	-75	10	Urban forest	12	13	1070	903	13	869	36346	0.49	4.34	70.07	46.15	83.33	15
104	Philadelphia	40	-75	15	Urban forest	12	13	1070	903	13	869	36346	0.49	4.34	44.66	64.89	82.25	99
105	Phoenix	33	-112	10	Urban lawn	355	24	194	781	48	386	32038	0.25	0.10	18.68	24.01	43.35	100
106	Phoenix	33	-112	20	Urban forest	355	24	194	781	48	386	32038	0.25	0.10	10.00	24.80	24.80	101
107	Porto	41	-9	20	Urban forest	94	15	1228	394	54	1297	13854	0.46	1.84	32.48	72.58	72.58	68
108	Potchefstroom	-27	27	10	Urban lawn	1100	15	600	464	75	93	7496	0.38	-2.40	55.70	46.45	83.87	18
109	Coahuila	26	-101	15	Urban forest	1403	16	350	405	60	313	10873	0.20	2.94	26.10	46.79	59.30	102
110	Registro	-24	-46	10	Urban forest	31	23	1563	237	41	2266	7098	0.43	2.04	12.93	17.76	32.06	103
111	Salzburg	48	13	20		436	9	1169	727	37	174	24909	0.49	3.01	41.60	83.70	83.70	24
112	San Juan	18	-66	10	Urban forest	30	26	1750	118	27	630	27938	0.44	2.32	33.10	35.42	63.94	104
113	Santiago de Compostela	42	-9	20	Urban forest	260	13	1787	393	46	440	16767	0.48	1.66	44.00	80.96	80.96	105
114	Seoul	37	127	10	Urban forest	40	13	1450	998	92	5060	13511	0.35	1.99	20.95	23.26	41.99	106
115	Sevilla	37	-6	10	Urban lawn	9	18	540	556	67	381	16731	0.27	-0.05	27.00	31.97	57.72	107
116	Sevilla	37	-6	20		9	18	540	556	67	381	16731	0.27	-0.05	20.00	50.78	50.78	14
117	Shanghai	31	121	20	Urban forest	4	16	1208	856	47	7658	14363	0.35	5.02	12.44	32.59	32.59	108
118	Shanghai	31	121	10	Urban forest	4	16	1208	856	47	7658	14363	0.35	5.02	12.85	16.06	29.00	109
119	Shanghai	31	121	10	Urban forest	4	16	1208	856	47	7658	14363	0.35	5.02	13.00	17.55	31.69	110
120	Shanghai	31	121	20	Urban forest	4	16	1208	856	47	7658	14363	0.35	5.02	18.98	53.14	53.14	111

ID	City	Lat	Lon	Depth	Vegetation	Ele	MAT	MAP	TS	PS	PD	GDPP	UGI	UHI	SOCC	SOCD	SOCD20	Reference
121	Shenyang	42	123	20	Urban forest	55	8	700	1292	97	2701	11348	0.29	5.05	30.54	67.19	67.19	112
122	Shenzhen	23	114	20		20	22	1924	540	76	5063	26071	0.30	1.98	9.16	23.83	23.83	113
123	Shenzhen	23	114	20		20	22	1924	540	76	5063	26071	0.30	1.98	14.00	37.52	37.52	114
124	Singapore	1	104	10	Urban lawn	20	28	2166	51	23	5311	52383	0.49	1.91	17.80	19.58	35.35	115
125	Singapore	1	104	20	Urban forest	20	28	2166	51	23	5311	52383	0.49	1.91	15.45	33.99	33.99	116
126	Singapore	1	104	10	Urban forest	20	28	2166	51	23	5311	52383	0.49	1.91	15.00	19.50	35.21	16
127	Smederevo	43	22	10	Urban forest	77	12	700	802	15	79	4374	0.45	0.85	26.80	31.80	57.41	25
128	Sochi	44	40	10	Urban lawn	26	17	1361	633	26	114	11598	0.49	2.49	53.10	45.97	82.99	65
129	Sofia	43	23	20	Urban forest	550	13	630	802	28	483	25565	0.43	3.42	25.00	60.43	60.43	117
130	Stoke-on-Trent	53	-2	15	Urban lawn	100	10	800	447	13	1114	16450	0.46	1.72	73.00	68.21	86.45	118
131	Strasbourg	49	8	20	Urban forest	154	11	352	658	28	406	17734	0.45	2.15	26.10	62.39	62.39	68
132	Stuttgart	49	9	20	Urban forest	207	11	665	667	31	1048	24532	0.49	2.65	54.10	92.35	92.35	119
133	Suwon	36	129	15		50	11	1261	839	66	547	28777	0.27	3.52	16.03	27.89	35.35	120
134	Székesfehérvár	47	18	10	Urban forest	150	10	540	811	25	119	10238	0.46	1.22	34.11	37.43	67.58	121
135	Székesfehérvár	47	18	20	Urban forest	150	10	540	811	25	119	10238	0.46	1.22	25.12	60.63	60.63	121
136	Taiyuan	38	113	20		800	10	420	1060	98	1737	7161	0.25	4.37	13.38	39.34	39.34	122
137	Tel Aviv	32	35	10		67	19	530	515	114	3007	22261	0.30	-1.96	17.05	22.26	40.18	123
138	Tel Aviv	32	35	10	Urban forest	67	19	530	515	114	3007	22261	0.30	-1.96	14.60	19.50	35.20	124
139	Tel Aviv	32	35	10	Urban forest	67	19	530	515	114	3007	22261	0.30	-1.96	10.15	14.11	25.47	125
140	Thessaloniki	41	23	20	Urban forest	120	15	352	764	24	457	12067	0.30	0.48	14.00	42.00	42.00	126
141	Thessaloniki	41	23	20		120	15	352	764	24	457	12067	0.30	0.48	9.20	25.78	25.78	24
142	Tianjin	39	117	20		10	14	585	1079	134	3790	17505	0.30	4.55	6.28	15.83	15.83	127
143	Tianjin	39	117	20	Urban forest	10	14	585	1079	134	3790	17505	0.30	4.55	10.10	26.26	26.26	128
144	Tokyo	36	140	5	Urban forest	40	14	1600	777	46	8955	20901	0.33	5.61	52.90	22.96	78.66	129
145	Tokyo	36	140	10	Urban forest	40	14	1600	777	46	8955	20901	0.33	5.61	51.80	45.66	82.44	129
146	Tokyo	36	140	20	Urban forest	40	14	1600	777	46	8955	20901	0.33	5.61	46.50	88.00	88.00	129

ID	City	Lat	Lon	Depth	Vegetation	Ele	MAT	MAP	TS	PS	PD	GDPP	UGI	UHI	SOCC	SOCD	SOCD20	Reference
147	Tokyo	36	140	5	Urban lawn	40	14	1600	777	46	8955	20901	0.33	5.61	55.20	23.19	79.43	129
148	Tokyo	36	140	10	Urban lawn	40	14	1600	777	46	8955	20901	0.33	5.61	52.45	45.82	82.73	129
149	Tokyo	36	140	20	Urban lawn	40	14	1600	777	46	8955	20901	0.33	5.61	45.83	87.48	87.48	129
150	Torreon	53	19	15		50	8	535	751	40	201	9725	0.43	2.15	30.00	51.64	65.45	76
151	Turin	45	8	20		18	17	598	708	38	781	18845	0.43	4.17	20.50	51.80	51.80	14
152	Uppsala	60	18	20		21	7	556	766	29	226	18817	0.46	1.94	31.00	70.39	70.39	14
153	Wulumqi	44	88	20		800	7	294	1449	38	1645	6710	0.27	1.71	6.00	17.28	17.28	130
154	Varanasi	25	83	15	Urban forest	76	30	1000	591	139	1249	2828	0.40	0.02	3.60	7.51	9.51	131
155	Varanasi	25	83	15	Urban forest	76	30	1000	591	139	1249	2828	0.40	0.02	7.90	13.27	16.82	132
156	Varanasi	25	83	10	Urban forest	76	30	1000	591	139	1249	2828	0.40	0.02	19.90	27.68	49.98	133
157	Varanasi	25	83	10	Urban forest	76	30	1000	591	139	1249	2828	0.40	0.02	19.40	33.17	59.89	133
158	Warsaw	52	21	20	Urban lawn	112	9	680	765	36	700	21183	0.48	1.50	21.70	54.20	54.20	134
159	Warsaw	52	21	20	Urban lawn	112	9	680	765	36	700	21183	0.48	1.50	20.50	51.80	51.80	135
160	Wuhu	31	118	20	Urban forest	15	16	1200	915	52	853	6122	0.29	2.85	11.00	30.80	30.80	136
161	Xi'an	34	109	20		400	15	545	943	64	2717	7566	0.29	1.72	13.59	35.88	35.88	137
162	Yan'an	37	109	20	Urban forest	1100	9	500	1028	92	126	4771	0.33	1.53	10.48	29.04	29.04	138
163	Zhengzhou	35	114	10	Urban forest	90	16	690	983	86	2453	7263	0.27	4.53	4.27	4.87	8.79	139
164	Zurich	47	9	20		556	12	550	665	23	706	40722	0.52	2.43	50.90	107.91	107.91	140
165	Glasgow	56	-4	15	Urban lawn	44	9	900	415	30	821	16967	0.47	2.02	55.00	69.51	88.10	118
166	Liverpool	53	-3	15	Urban lawn	50	9	796	445	20	943	16099	0.43	1.93	38.51	74.52	94.45	141
167	Knoxville	36	-84	10	Urban forest	298	14	1602	809	14	125	28986	0.50	3.15	33.06	45.95	82.97	142
168	Akure	7	5	20	Urban lawn	357	27	1453	117	66	524	5916	0.37	2.34	25.08	83.28	83.28	143
169	Okitipupa	6	5	20		32	28	1719	117	65	295	6219	0.44	-0.80	22.71	79.84	79.84	143
170	Akure	7	5	20	Urban lawn	357	27	1453	117	66	524	5916	0.37	2.34	18.55	61.60	61.60	143
171	Okitipupa	6	5	20		32	28	1719	117	65	295	6219	0.44	-0.80	18.41	66.04	66.04	143
172	Wulumqi	44	88	5	Urban lawn	800	7	294	1449	38	1645	6710	0.27	1.71	18.13	11.72	40.13	144

ID	City	Lat	Lon	Depth	Vegetation	Ele	MAT	MAP	TS	PS	PD	GDPP	UGI	UHI	SOCC	SOCD	SOCD20	Reference
173	Peshawar	34	72	15		170	25	428	817	57	977	4277	0.30	0.47	32.50	54.45	69.02	145
174	Ancona	44	14	5		152	14	1111	651	20	852	13254	0.40	1.02	34.35	18.80	64.40	146
175	Guangzhou	22	113	20		22	21	1720	539	74	5063	22218	0.43	1.98	9.12	24.10	24.10	147
176	Guangzhou	22	113	20		22	21	1720	539	74	5063	22218	0.43	1.98	10.43	27.50	27.50	147
177	Piracicaba	-23	-48	5		132	28	913	256	72	194	374	0.43	1.77	25.00	15.11	51.75	148
178	Santo Amaro	-13	-39	5		364	23	739	140	42	288	10175	0.41	1.27	25.00	15.11	51.75	148
179	Río Piedras [San Juan]	18	-66	10	Urban forest	82	24	1972	118	27	630	27938	0.44	2.32	34.00	34.00	61.39	149
180	Vilnius	55	25	5	Urban forest	130	9	735	835	27	331	21361	0.47	1.56	35.34	19.13	65.52	150
181	Glasgow	56	-4	10		44	9	900	415	30	821	16967	0.47	2.02	63.80	46.90	84.68	151
182	Glasgow	56	-4	20		44	9	900	415	30	821	16967	0.47	2.02	57.13	93.29	93.29	151
183	Aveiro	40	-8	10		16	18	1000	438	51	114	12720	0.38	1.45	9.28	12.99	23.46	151
184	Aveiro	40	-8	20		16	18	1000	438	51	114	12720	0.38	1.45	8.27	23.35	23.35	151
185	Uppsala	60	18	20	Urban lawn	21	7	556	766	29	226	18817	0.46	1.94	39.45	89.94	89.94	152
186	Malmö	56	13	20	Urban lawn	9	8	540	642	19	303	17839	0.42	1.30	32.53	67.01	67.01	152
187	Gothenburg	58	12	20	Urban lawn	34	7	714	671	20	611	19410	0.45	1.20	40.87	75.20	75.20	152
188	Santiago de Compostela	42	-9	10	Urban forest	260	13	1787	393	46	440	16767	0.48	1.66	34.80	37.90	68.42	153
189	Santiago de Compostela	42	-9	10	Urban lawn	260	13	1787	393	46	440	16767	0.48	1.66	31.29	35.41	63.93	153
190	Székesfehérvár	47	18	10	Urban forest	200	10	540	811	25	119	10238	0.46	1.22	58.80	46.81	84.52	121
191	Székesfehérvár	47	18	20	Urban forest	200	10	540	811	25	119	10238	0.46	1.22	51.05	90.94	90.94	121
192	Székesfehérvár	47	18	10		200	10	540	811	25	119	10238	0.46	1.22	39.90	40.97	73.96	121
193	Székesfehérvár	47	18	20		200	10	540	811	25	119	10238	0.46	1.22	39.65	81.66	81.66	121
194	Székesfehérvár	47	18	10		200	10	540	811	25	119	10238	0.46	1.22	65.20	46.82	84.53	121
195	Székesfehérvár	47	18	20		200	10	540	811	25	119	10238	0.46	1.22	55.30	92.78	92.78	121
196	Atlanta	34	-84	20		304	17	1416	748	15	983	32075	0.52	3.24	44.70	86.55	86.55	154
197	Camden	40	-75	20		3	13	1100	897	13	869	36346	0.49	4.34	73.00	90.94	90.94	154
198	Cincinnati	39	-84	20		221	12	1100	933	17	404	29811	0.51	3.08	42.60	84.67	84.67	154

ID	City	Lat	Lon	Depth	Vegetation	Ele	MAT	MAP	TS	PS	PD	GDPP	UGI	UHI	SOCC	SOCD	SOCD20	Reference
199	Cleveland	41	-82	20		246	10	979	950	18	565	30779	0.51	3.76	31.20	70.69	70.69	154
200	Detroit	42	-83	20		203	10	881	995	19	875	29087	0.51	4.35	31.30	70.84	70.84	154
201	New Orleans	30	-90	20		3	21	1645	616	16	246	34816	0.43	1.47	26.80	63.60	63.60	154
202	Omaha	41	-96	20		343	11	777	1105	53	229	33067	0.50	1.84	42.90	84.95	84.95	154
203	Phoenix	33	-112	20		355	24	194	781	48	386	32038	0.25	0.10	25.50	61.32	61.32	154
204	Portland	46	-123	20		70	12	1162	583	59	551	28792	0.49	4.07	48.00	89.08	89.08	154
205	Río Piedras [San Juan]	18	-66	20		33	24	1972	118	27	630	27938	0.44	2.32	66.20	93.46	93.46	154
206	Tacoma	47	-122	20		75	11	1233	506	59	689	32540	0.51	4.32	68.30	92.92	92.92	154
207	Panzhihua	27	102	20		1174	18	996	485	94	316	12229	0.37	2.98	14.00	37.60	37.60	155
208	Wroclaw	51	17	20	Urban lawn	125	10	548	731	43	259	10798	0.50	1.69	34.70	75.66	75.66	156
209	Caserta	41	14	5	Urban forest	46	10	938	612	44	267	20888	0.42	4.34	63.29	23.46	80.37	157
210	Wuhan	31	114	10	Urban lawn	7	16	1100	899	53	3228	18686	0.35	2.51	11.30	15.54	28.07	158
211	Campinas	-23	-47	10	Urban forest	754	21	1462	229	67	1007	17644	0.36	4.44	27.80	32.65	58.94	159
212	Campinas	-23	-47	20	Urban forest	754	21	1462	229	67	1007	17644	0.36	4.44	23.90	58.41	58.41	159
213	Naples [Italy]	41	14	5		5	15	908	605	49	2830	20421	0.39	2.51	45.53	15.50	53.11	160
214	Philadelphia	40	-75	10	Urban forest	32	13	1239	903	13	869	36346	0.49	4.34	80.04	42.98	77.60	161
215	Newark	40	-74	10	Urban forest	10	13	1140	861	10	621	28171	0.53	4.34	44.66	50.91	91.92	161
216	Bobo-Dioulasso	11	-4	15		450	27	1050	181	106	292	1191	0.33	1.76	19.00	36.53	46.30	162
217	Hartford	42	-73	10	Urban lawn	37	23	840	939	6	477	45003	0.52	4.34	24.91	28.90	52.18	163
218	Hartford	42	-73	20	Urban lawn	37	23	840	939	6	477	45003	0.52	4.34	19.39	47.30	47.30	163
219	Brno	49	17	10	Urban lawn	241	10	552	769	45	296	14718	0.44	1.80	63.50	46.91	84.70	164
220	Edmonton	54	-113	15	Urban lawn	675	4	348	1116	71	256	28849	0.39	1.58	43.00	63.78	80.84	165
221	Estarreja	41	-9	20		24	16	1295	375	55	349	12720	0.38	1.03	21.70	54.20	54.20	166
222	Viseu	41	-8	20		459	14	1251	501	59	80	12720	0.38	1.03	32.20	72.17	72.17	166
223	Lisbon	39	-9	20		86	18	877	405	63	1569	24257	0.36	1.72	57.60	93.40	93.40	166
224	Estarreja	41	-9	20		24	16	1295	375	55	349	12720	0.38	1.03	27.40	64.62	64.62	166

ID	City	Lat	Lon	Depth	Vegetation	Ele	MAT	MAP	TS	PS	PD	GDPP	UGI	UHI	SOCC	SOCD	SOCD20	Reference
225	Viseu	41	-8	20		459	14	1251	501	59	80	12720	0.38	1.03	15.20	40.37	40.37	166
226	Lisbon	39	-9	20		86	18	877	405	63	1569	24257	0.36	1.72	39.90	81.93	81.93	166
227	Novi Sad	45	20	10		82	13	643	783	30	230	3098	0.47	1.33	40.02	41.03	74.08	167
228	Novi Sad	45	20	10		82	13	643	783	30	230	3098	0.47	1.33	42.69	42.38	76.51	167
229	Salt Lake City	41	-112	10	Urban lawn	1363	11	397	949	31	465	26379	0.40	3.90	27.00	25.60	46.21	168
230	Salt Lake City	41	-112	20	Urban lawn	1363	11	397	949	31	465	26379	0.40	3.90	23.50	57.34	57.34	168
231	Lubbock	34	-102	10	Urban lawn	987	16	487	828	58	110	33476	0.35	-0.07	21.11	26.18	47.27	169
232	Budapest	47	19	10	Urban lawn	130	12	516	781	25	557	27860	0.45	1.97	25.40	30.57	55.20	170
233	Beijing	40	116	10	Urban forest	51	12	640	1098	133	6609	16241	0.33	4.90	8.10	11.46	20.69	171
234	Beijing	40	116	20	Urban forest	51	12	640	1098	133	6609	16241	0.33	4.90	7.56	21.49	21.49	171
235	Anji	31	120	10	Urban forest	24	14	1500	905	51	381	10432	0.34	2.12	14.80	18.35	33.13	172
236	Shanghai	31	121	20		4	16	1208	856	47	7658	14363	0.35	5.02	16.82	44.01	44.01	173
237	Brisbane	-27	153	15	Urban lawn	60	19	1110	374	60	797	29333	0.42	2.31	15.00	31.50	39.92	174
238	Tonghua	42	126	5	Urban forest	394	5	874	1344	97	233	6001	0.35	1.70	91.35	18.23	62.44	175
239	Baishan	42	126	5	Urban lawn	494	3	753	1347	96	230	7600	0.28	2.46	21.21	13.31	45.59	175
240	Yanji	43	129	5	Urban forest	204	6	550	1283	96	167	8484	0.31	3.68	22.96	14.16	48.51	175
241	Dunhua	43	128	5	Urban forest	511	4	628	1327	98	133	6898	0.32	2.55	61.74	23.47	80.40	175
242	Jilin	44	127	5	Urban forest	201	4	678	1426	102	793	7609	0.35	3.44	35.21	19.08	65.37	175
243	Santiago	-33	-71	5	Urban forest	115	15	491	476	102	1278	5013	0.31	0.96	28.24	16.50	56.54	175
244	Belo Horizonte	-20	-44	5	Urban lawn	721	21	1504	185	87	735	13039	0.36	2.63	21.11	13.26	45.42	175
245	Contagem	-20	-44	5	Urban forest	858	21	1577	184	88	735	13039	0.43	2.63	28.12	16.45	56.37	175
246	Betim	-20	-44	5	Urban lawn	830	21	1511	190	87	735	11111	0.36	2.63	12.55	8.53	29.23	175
247	Longmont	40	-105	5	Urban lawn	1531	10	428	877	47	162	30760	0.42	4.96	44.71	21.64	74.14	175
248	Grand Junction	39	-109	5	Urban lawn	1423	11	231	981	25	51	34131	0.37	0.03	13.90	9.34	32.00	175
249	Cheyenne	41	-105	5	Urban lawn	1852	8	389	849	57	40	35042	0.39	-0.49	21.38	13.39	45.88	175
250	South Lyon	42	-84	5	Urban lawn	283	9	814	998	20	217	29087	0.51	4.35	23.93	14.61	50.07	175

ID	City	Lat	Lon	Depth	Vegetation	Ele	MAT	MAP	TS	PS	PD	GDPP	UGI	UHI	SOCC	SOCD	SOCD20	Reference
251	Oxford	52	-1	5	Urban lawn	72	6	573	766	29	226	27175	0.49	1.94	101.19	14.12	48.36	175
252	Bodø	67	14	5	Urban lawn	8	2	1537	554	30	14	20056	0.51	0.55	65.41	23.40	80.17	175
253	Uppsala	60	18	5	Urban lawn	21	7	556	766	29	226	18817	0.46	1.94	79.46	21.62	74.05	175
254	Poitiers	47	0	5	Urban forest	111	12	693	556	17	121	15507	0.49	2.02	44.50	21.59	73.98	175
255	Niort	46	0	5	Urban forest	40	12	825	551	26	80	15507	0.49	0.79	41.76	20.96	71.82	175
256	Tours	47	1	5	Urban forest	73	12	661	552	15	210	16001	0.45	1.51	20.10	12.74	43.66	175
257	Ljubljana	46	15	5	Urban forest	302	10	1452	768	25	161	22088	0.47	3.94	38.43	20.07	68.77	175
258	Koper	46	14	5	Urban forest	11	14	977	705	23	286	7369	0.24	0.54	57.74	23.36	80.02	175
259	Maribor	47	16	5	Urban forest	273	10	1045	743	31	147	12078	0.44	1.94	34.75	18.93	64.85	175
260	Pretoria	-26	28	5	Urban forest	1365	18	708	392	82	1213	10100	0.41	0.14	33.33	18.45	63.19	175
261	Germiston	-26	28	5	Urban lawn	1644	16	725	403	79	1879	10915	0.39	0.14	31.07	17.62	60.37	175
262	Cape Town	-34	18	5	Urban forest	48	17	538	292	68	782	8001	0.35	0.45	57.86	23.36	80.04	175
263	Durgapur	24	87	5	Urban forest	585	26	1298	446	105	809	5310	0.41	-0.30	12.69	8.62	29.54	175
264	Mirzapur	25	83	5	Urban lawn	309	26	974	603	145	1851	4021	0.43	0.71	29.42	16.98	58.18	175
265	Agra	27	78	5	Urban lawn	1584	26	802	684	131	1558	3023	0.48	-3.75	10.87	7.51	25.72	175
266	Beijing	40	116	5	Urban forest	51	12	640	1098	133	6609	16241	0.33	4.90	12.20	8.33	28.52	175
267	Tai'an	36	117	5	Urban forest	80	12	544	1043	111	510	5966	0.35	2.71	11.18	7.70	26.37	175
268	Tianjin	39	117	5	Urban forest	6	13	576	1079	134	3790	17505	0.30	4.55	16.53	10.84	37.14	175
269	Wulumqi	44	88	5	Urban lawn	800	7	210	1449	38	1645	6710	0.27	1.71	55.12	23.18	79.41	175
270	Alice Springs	-24	134	5	Urban forest	562	21	329	626	50	11	23527	0.44	-0.83	15.66	10.36	35.48	175
271	Brisbane	-27	153	5	Urban lawn	34	21	1190	374	60	797	29333	0.42	4.08	69.68	23.11	79.18	175
272	Mildura	-34	142	5	Urban lawn	60	17	301	535	15	24	25729	0.34	-3.55	35.83	19.28	66.06	175
273	Sydney	-34	151	5	Urban lawn	48	17	910	429	30	596	30159	0.43	5.08	47.77	22.23	76.15	175
274	Barcelona	41	2	5	Urban forest	32	16	653	512	32	6792	14085	0.28	2.46	27.25	16.09	55.13	175
275	Pullman	47	-117	5	Urban forest	717	9	531	745	38	25	31465	0.39	0.77	35.11	19.05	65.26	175
276	Mexico City	19	-99	5	Urban forest	2316	15	867	182	94	3532	18488	0.26	7.40	44.80	21.66	74.20	175

ID	City	Lat	Lon	Depth	Vegetation	Ele	MAT	MAP	TS	PS	PD	GDPP	UGI	UHI	SOCC	SOCD	SOCD20	Reference
277	Mexico City	19	-99	5	Urban forest	2316	16	796	182	94	3532	18488	0.26	7.40	56.48	23.28	79.76	175
278	Madrid	40	-4	5	Urban forest	41	15	428	667	40	1722	20550	0.21	2.15	21.08	13.24	45.36	175
279	Imessa Odo	8	5	5	Urban forest	270	25	1321	118	66	770	3387	0.40	3.44	6.36	4.56	15.63	175
280	Imessa Odo	8	5	5	Urban forest	270	26	1371	118	66	770	3387	0.40	3.44	17.94	11.61	39.78	175
281	Imessa Odo	7	5	5	Urban forest	270	26	1399	118	66	482	3387	0.40	3.44	9.86	6.87	23.52	175
282	Lakeland	28	-82	5	Urban forest	55	22	1269	448	53	209	33240	0.50	1.56	20.68	13.04	44.67	175
283	Utrera	37	-6	5	Urban forest	49	18	580	571	67	381	4062	0.21	1.62	7.56	5.37	18.40	175
284	Coimbra	40	-8	5	Urban forest	75	16	934	438	52	114	12720	0.38	1.45	7.37	5.25	17.98	175
285	Porto	41	-9	5	Urban forest	83	15	1228	394	54	1297	13854	0.46	1.84	33.86	18.63	63.82	175
286	Jerusalem	32	35	5	Urban forest	730	16	439	586	109	972	11836	0.24	-1.96	58.37	23.39	80.13	175
287	Beer Sheva	31	35	5	Urban forest	260	19	216	562	105	160	22268	0.19	-1.96	18.37	11.84	40.58	175
288	Ofakim	31	35	5	Urban forest	149	20	245	543	107	160	17927	0.27	-1.96	14.79	9.86	33.78	175
289	Ningbo	30	122	10		6	17	1569	827	41	1830	14155	0.30	4.86	15.40	19.25	34.76	176
290	Nanjing	32	119	10	Urban forest	21	16	1091	930	58	2687	15062	0.31	2.83	15.90	20.98	37.88	177
291	Nanjing	32	119	20	Urban forest	21	16	1091	930	58	2687	15062	0.31	2.83	13.00	35.23	35.23	177
292	Yancheng	33	120	5	Urban forest	3	14	1051	931	71	655	16753	0.34	3.26	12.78	8.68	29.73	178
293	Yancheng	33	120	5	Urban lawn	3	14	1051	931	71	655	16753	0.34	3.26	15.31	10.16	34.80	178
294	Yancheng	33	120	5	Urban lawn	3	14	1051	931	71	655	16753	0.34	3.26	14.44	9.66	33.08	178
295	Mendoza	-33	-69	20	Urban forest	772	17	188	599	67	304	13732	0.25	-1.46	19.14	49.00	49.00	179
296	Xiamen	25	118	10	Urban forest	24	21	1137	598	59	1855	13116	0.30	3.26	15.24	20.23	36.53	180
297	Xiamen	25	118	10	Urban lawn	24	21	1137	598	59	1855	13116	0.30	3.26	15.74	20.80	37.56	180
298	Sosnowiec	50	19	20	Urban forest	272	9	812	756	40	645	10596	0.51	2.12	32.00	71.88	71.88	181
299	Changsha	28	113	20	Urban forest	58	17	1448	869	48	897	10579	0.31	0.42	13.10	35.47	35.47	182
300	Changsha	28	113	20	Urban lawn	58	17	1448	869	48	897	10579	0.31	0.42	11.80	32.32	32.32	182
301	Hong Kong	22	114	5	Urban lawn	65	23	2147	540	76	8243	43639	0.37	1.98	24.66	14.95	51.22	183
302	Hong Kong	22	114	10	Urban lawn	65	23	2147	540	76	8243	43639	0.37	1.98	18.34	23.65	42.71	183

ID	City	Lat	Lon	Depth	Vegetation	Ele	MAT	MAP	TS	PS	PD	GDPP	UGI	UHI	SOCC	SOCD	SOCD20	Reference
303	Hong Kong	22	114	15	Urban lawn	65	23	2147	540	76	8243	43639	0.37	1.98	14.65	26.36	33.41	183
304	Baltimore	39	-77	10	Urban forest	70	13	1154	897	11	939	32721	0.47	2.86	44.20	43.06	77.75	46
305	Baltimore	39	-77	10	Urban lawn	70	13	1154	897	11	939	32721	0.47	2.86	32.50	36.30	65.54	46
306	Baltimore	39	-77	10	Urban forest	70	13	1154	897	11	939	32721	0.47	2.86	43.67	46.72	84.36	184
307	Baltimore	39	-77	10	Urban lawn	70	13	1154	897	11	939	32721	0.47	2.86	46.00	50.60	91.36	184
308	Padang	-1	100	20		44	29	3552	28	20	508	5496	0.47	3.27	26.80	48.78	48.78	185
309	Tangshan	40	118	20		31	12	600	1111	126	1224	8567	0.36	2.09	11.98	35.69	35.69	186
310	Xuzhou	34	117	20		43	15	841	986	90	1008	14540	0.36	2.46	22.38	56.40	56.40	187
311	Hengshui	38	116	20		22	14	546	1129	121	557	7330	0.30	1.55	12.09	33.62	33.62	188
312	Qiqihar	47	124	20		146	3	415	1497	115	534	5771	0.30	3.46	9.85	26.41	26.41	189
313	Khartoum	16	33	20		382	30	113	412	153	491	2220	0.15	2.44	6.70	23.58	23.58	190
314	Yilan	25	122	15		11	22	2700	450	29	583	23143	0.36	6.74	17.70	34.45	43.66	191
315	Taipei	25	121	15		100	22	2100	509	29	4482	34497	0.32	3.81	18.13	35.14	44.54	191
316	Kaohsiung	23	120	15		15	24	1700	376	106	2295	35055	0.32	3.81	4.10	9.00	11.41	191
317	Edinburgh	56	-3	20		59	8	962	410	14	399	17083	0.46	2.02	63.16	93.85	93.85	192
318	Leeds	54	-2	20		111	9	1138	457	17	1102	15762	0.48	2.15	79.93	86.06	86.06	192
319	Liverpool	53	-3	20		35	10	955	445	20	943	16099	0.43	1.93	56.25	93.07	93.07	192
320	Nottingham	53	-1	20		56	10	757	474	11	1016	16931	0.44	2.15	75.99	89.13	89.13	192
321	Leicester	53	-1	20		81	10	757	479	10	1016	15603	0.43	2.15	48.36	89.32	89.32	192
322	Milton Keynes	52	-1	20		86	10	704	479	11	506	24767	0.53	2.13	34.54	75.44	75.44	192
323	Cardiff	51	-3	20		30	11	1108	450	24	444	14669	0.45	0.71	99.67	59.31	59.31	192
324	Bristol	51	-3	20		52	10	1095	462	19	506	17758	0.45	0.71	66.12	93.47	93.47	192
325	Southampton	51	-1	20		23	11	976	458	26	1016	25285	0.46	1.88	39.47	81.47	81.47	192
326	Oskemen	50	83	20	Urban lawn	293	4	549	1350	21	30	17881	0.45	0.31	33.06	73.40	73.40	193
327	Chelyabinsk	55	61	10	Urban lawn	168	7	602	1246	55	613	6256	0.51	1.51	28.30	33.06	59.69	65
328	Dijon	47	5	20		300	11	982	645	16	165	17041	0.42	2.56	34.90	76.78	76.78	194

ID	City	Lat	Lon	Depth	Vegetation	Ele	MAT	MAP	TS	PS	PD	GDPP	UGI	UHI	SOCC	SOCD	SOCD20	Reference
329	Ulan-Ude	52	108	20	Urban forest	219	5	306	1547	109	195	8446	0.37	4.40	42.00	84.09	84.09	195
330	Ulan-Ude	52	108	20	Urban forest	219	5	306	1547	109	195	8446	0.37	4.40	48.00	89.08	89.08	195
331	São Paulo	-24	-47	20	Urban forest	800	27	598	237	59	4322	3906	0.37	4.44	21.20	53.21	53.21	196
332	Apatity	68	33	10	Urban forest	152	0	853	983	35	22	5593	0.44	0.51	40.82	40.00	72.22	197
333	Bratislava	48	17	10		155	11	663	772	25	309	32758	0.48	-0.01	21.90	27.29	49.28	198
334	Xiamen	25	118	5	Urban lawn	24	22	1464	598	59	1855	13116	0.30	3.26	30.00	17.21	58.97	199
335	Mashhad	36	60	20		1011	14	229	862	80	377	8090	0.17	-0.59	32.50	72.61	72.61	200
336	Singapore	1	104	10	Urban lawn	21	28	2154	51	23	5311	52383	0.49	1.91	12.50	17.01	30.72	201
337	Bydgoszcz	53	18	20		48	10	564	746	38	233	10436	0.49	1.05	25.80	61.85	61.85	202
338	Teresina	-5	-43	10		41	26	1478	115	92	353	590	0.36	1.34	10.90	15.05	27.18	203
339	Wroclaw	51	17	20	Urban lawn	193	9	583	731	43	259	10798	0.50	1.69	58.24	93.53	93.53	204
340	Manaus	-3	-60	20		638	26	2300	53	45	495	15031	0.38	1.09	6.60	18.92	18.92	205
341	Lagos	7	3	10		24	28	1342	122	70	3423	6440	0.28	2.80	10.44	14.47	26.13	206
342	Hohhot	41	112	20		1069	7	386	1253	108	979	9088	0.27	3.36	14.91	38.76	38.76	207
343	Hohhot	41	112	20		1069	7	386	1253	108	979	9088	0.27	3.36	12.99	36.12	36.12	207
344	Kunming	25	103	20		1923	15	1000	476	84	1079	5057	0.27	2.61	9.53	26.64	26.64	208
345	Linfen	36	112	20		450	11	527	1048	88	581	6549	0.30	2.31	6.09	17.53	17.53	209
346	Khorramabad	33	48	20		1222	15	360	881	87	45	4782	0.18	0.86	22.88	56.47	56.47	210
347	Havana	23	-82	20		53	26	1341	243	47	1975	16130	0.43	3.26	31.86	71.67	71.67	211
348	Çanakkale	40	26	5		23	16	786	695	62	76	10597	0.30	1.10	22.62	14.00	47.95	212
349	Jagiroad	26	92	20		76	26	1399	424	91	633	1119	0.40	0.71	25.60	61.50	61.50	213
350	Manchester	53	-2	10	Urban lawn	92	10	1010	449	19	1114	16084	0.47	1.93	64.50	55.47	100.14	214
351	Shanghai	31	121	20		4	16	1208	856	47	7658	14363	0.35	5.02	24.94	60.32	60.32	215
352	Mexico City	19	-99	10	Urban forest	2316	17	586	182	94	3532	18488	0.26	7.40	26.00	22.62	40.84	216
353	New York	41	-74	10		38	13	1161	893	10	6041	36082	0.49	4.34	54.70	46.28	83.57	217
354	Madison	43	-89	15	Urban lawn	267	18	1300	1077	39	191	30554	0.52	1.39	33.06	65.46	82.97	218

ID	City	Lat	Lon	Depth	Vegetation	Ele	MAT	MAP	TS	PS	PD	GDPP	UGI	UHI	SOCC	SOCD	SOCD20	Reference
355	Pinellas	28	-83	15		5	29	1240	461	55	1178	38117	0.49	1.56	25.00	45.32	57.44	219
356	Santiago de Compostela	43	-9	10		253	13	1242	393	46	440	16767	0.48	1.66	42.70	42.38	76.52	220
357	Kumasi	7	-2	15		258	26	1250	107	53	1288	2935	0.35	4.92	13.28	27.89	35.35	64
358	Qingdao	36	120	10		30	13	604	977	90	2471	11327	0.25	3.26	34.51	37.70	68.07	221
359	Baoding	39	115	20		20	13	500	1119	129	962	7857	0.35	2.24	14.13	37.90	37.90	222
360	Guiyang	27	107	20	Urban forest	1185	15	1200	681	70	1195	4061	0.31	1.64	30.40	69.47	69.47	223
361	Tianshui	35	106	10		1213	9	552	880	80	471	9023	0.30	2.44	5.90	8.50	15.36	224
362	Wuxi	32	120	10	Urban forest	15	17	1393	906	54	2726	10790	0.31	4.34	19.93	28.30	51.10	225
363	Harbin	46	127	20	Urban forest	146	4	529	1486	107	2495	6578	0.32	3.82	27.99	78.37	78.37	226
364	Harbin	46	127	20	Urban forest	146	4	529	1486	107	2495	6578	0.32	3.82	19.87	54.45	54.45	227
365	Neuchatel	47	7	10		1192	9	1456	647	16	334	29332	0.54	2.43	52.00	31.20	56.33	228
366	Manchester_USA	43	-71	20	Urban lawn	83	10	1132	993	10	483	31530	0.50	2.77	30.40	58.37	58.37	229
367	Lubbock	34	-102	15	Urban lawn	990	17	450	828	58	110	33476	0.35	-0.07	28.16	50.69	64.24	230
368	Albuquerque	35	-107	15	Urban lawn	1596	12	354	858	52	335	30185	0.26	-0.59	19.30	37.00	46.89	231
369	Atlanta	34	-84	15	Urban lawn	304	17	1416	748	15	983	32075	0.52	3.24	14.30	28.72	36.40	231
370	Cheyenne	41	-105	15	Urban lawn	1852	8	412	849	57	40	35042	0.39	-0.49	21.70	40.65	51.52	231
371	Dallas	33	-97	15	Urban lawn	182	19	1023	822	31	1019	35747	0.43	-0.34	22.90	42.39	53.73	231
372	Denver	40	-105	15	Urban lawn	1673	10	413	865	43	700	35796	0.43	4.96	31.70	53.58	67.91	231
373	Duluth	47	-92	15	Urban lawn	206	5	804	1141	50	85	33373	0.46	2.03	28.36	49.67	62.95	231
374	Houston	30	-95	15	Urban lawn	26	21	1264	640	21	1154	36992	0.48	1.66	21.49	40.34	51.12	231
375	Las Vegas	36	-115	15	Urban lawn	675	19	153	896	44	952	32418	0.19	2.06	25.09	45.44	57.59	231
376	Minneapolis [Saint Paul]	45	-93	15	Urban lawn	270	8	843	1517	51	707	33597	0.56	2.03	51.68	68.45	86.76	231
377	Orlando	29	-81	15	Urban lawn	31	23	1499	454	51	583	37103	0.49	1.56	17.98	34.90	44.23	231
378	Phoenix	33	-112	15	Urban lawn	355	24	194	781	48	386	32038	0.25	0.10	21.13	39.80	50.44	231
379	Portland	46	-123	15	Urban lawn	70	12	1162	583	59	551	28792	0.49	4.07	21.21	39.92	50.60	231
380	San Francisco	38	-122	15	Urban lawn	16	19	928	269	92	1759	13778	0.31	1.94	27.69	48.83	61.89	231

ID	City	Lat	Lon	Depth	Vegetation	Ele	MAT	MAP	TS	PS	PD	GDPP	UGI	UHI	SOCC	SOCD	SOCD20	Reference
381	Seattle	47	-122	15	Urban lawn	80	12	1143	520	57	689	32540	0.51	4.32	25.52	46.02	58.33	231
382	Wichita	38	-97	15	Urban lawn	408	15	847	1006	45	219	33402	0.47	-0.09	21.96	41.03	52.00	231
383	Wooster	41	-82	15	Urban lawn	303	11	1081	947	21	104	31553	0.53	0.90	32.02	53.93	68.35	231
384	Baltimore	39	-77	10	Urban lawn	70	13	1154	897	11	939	32721	0.47	2.86	21.85	23.69	42.76	232
385	Boston	42	-71	10	Urban lawn	28	11	1169	926	8	1094	43495	0.52	2.77	23.69	20.61	37.20	232
386	Los Angeles	34	-118	10	Urban lawn	173	18	236	392	100	1404	39525	0.29	0.64	23.06	17.69	31.94	232
387	Miami	26	-80	10	Urban lawn	8	25	1885	328	45	875	38891	0.43	3.04	31.40	28.79	51.98	232
388	Minneapolis [Saint Paul]	45	-93	10	Urban lawn	270	8	843	1517	51	707	33597	0.56	2.03	28.38	29.52	53.29	232
389	Phoenix	33	-112	10	Urban lawn	355	24	194	781	48	386	32038	0.25	0.10	10.38	11.00	19.87	232
390	Melbourne	-38	145	10	Urban lawn	63	15	647	375	14	964	27030	0.40	1.96	34.00	41.48	74.89	233
391	Aveiro	40	-8	10		16	18	1000	438	51	114	12720	0.38	1.45	19.00	24.35	43.97	234
392	Ljubljana	46	15	10		302	10	1452	768	25	161	22088	0.47	3.94	53.00	45.95	82.95	234
393	Turin	45	8	10		18	17	598	708	38	781	18845	0.43	4.17	21.00	26.40	47.67	234
394	Uppsala	60	18	10		21	7	556	766	29	226	18817	0.46	1.94	42.00	42.05	75.91	234
395	Ljubljana	46	15	20		302	10	1452	768	25	161	22088	0.47	3.94	43.35	85.37	85.37	235
396	Seville	37	-6	20		173	16	510	556	67	381	22576	0.33	-0.05	15.50	41.06	41.06	235
397	Turin	45	8	20		18	17	598	708	38	781	18845	0.43	4.17	17.60	45.72	45.72	235
398	Nanchang	29	116	20	Urban forest	25	18	1700	866	60	1520	6467	0.38	2.81	15.02	41.76	41.76	236
399	Vancouver	49	-123	15	Urban forest	55	12	1189	548	52	818	22688	0.43	5.78	41.24	62.50	79.22	237
400	Madrid	40	-4	20		41	18	261	667	40	1722	20550	0.21	2.15	26.86	63.70	63.70	238
401	Los Angeles	34	-118	5	Urban lawn	173	18	236	392	100	1404	39525	0.29	0.64	77.14	22.08	75.63	239
402	Baltimore	39	-77	10	Urban lawn	70	13	1154	897	11	939	32721	0.47	2.86	40.60	32.07	57.91	240
403	Puducherry	12	80	20	Urban forest	431	24	1297	239	99	1466	467	0.39	-0.45	7.50	19.50	19.50	241
404	Puducherry	12	80	20	Urban lawn	431	24	1297	239	99	1466	467	0.39	-0.45	7.00	19.60	19.60	241
405	Tezpur	27	93	10	Urban lawn	48	23	2524	423	90	444	6648	0.44	1.71	12.60	18.27	32.99	242
406	Tezpur	27	93	10	Urban forest	48	23	2524	423	90	444	6648	0.44	1.71	15.60	21.37	38.59	242

ID	City	Lat	Lon	Depth	Vegetation	Ele	MAT	MAP	TS	PS	PD	GDPP	UGI	UHI	SOCC	SOCD	SOCD20	Reference
407	Delhi [New Delhi]	29	77	10	Urban lawn	216	25	1065	694	135	7721	2220	0.40	2.43	12.80	17.37	31.37	243
408	Shihezi	44	86	10		400	8	230	1520	44	241	7961	0.36	1.31	15.49	20.51	37.03	244
409	Bucharest	44	26	20		80	13	691	877	35	1020	28720	0.40	1.74	17.40	45.28	45.28	245
410	Iassy	48	27	20		43	12	555	852	50	59	4908	0.44	2.74	18.00	46.58	46.58	245
411	Baia Mare	48	24	20		227	8	796	804	28	68	5257	0.40	4.27	8.80	24.75	24.75	245
412	Seoul	37	127	15	Urban forest	54	12	1115	998	92	5060	13511	0.35	1.99	8.70	19.31	24.48	246
413	Suwon	36	129	15	Urban forest	50	11	1261	839	66	547	28777	0.27	3.52	4.79	8.84	11.20	246
414	Naples [Italy]	41	14	5	Urban forest	5	15	908	605	49	2830	20421	0.39	2.51	77.72	21.97	75.26	247
415	Abuja	9	7	10		436	27	1125	181	96	704	3877	0.35	0.53	7.50	10.67	19.26	248
416	Beijing	40	116	20		51	12	640	1098	133	6609	16241	0.33	4.90	9.59	26.47	26.47	249
417	Beijing	40	116	20		51	12	640	1098	133	6609	16241	0.33	4.90	10.22	29.02	29.02	249
418	Beijing	40	116	20	Urban forest	51	12	640	1098	133	6609	16241	0.33	4.90	9.57	24.50	24.50	249
419	Geneva	46	6	10	Urban lawn	418	11	985	670	14	558	44417	0.48	4.29	45.13	49.64	89.63	250
420	Toronto	44	-80	5		157	8	839	975	15	1154	27690	0.45	5.61	60.00	23.45	80.32	251

Supplementary References

1. Fick, S. E. & Hijmans, R. J. WorldClim 2: new 1-km spatial resolution climate surfaces for global land areas. *Int. J. Climatol.* **37**, 4302-4315 (2017).
2. Florczyk, A. J. et al. GHSL data package 2019. Publication Office of the European Union. (2019).
3. Corbane, C. et al. The grey-green divide: multi-temporal analysis of greenness across 10,000 urban centres derived from the Global Human Settlement Layer (GHSL). *International Journal of Digital Earth*. **13**, 101-118 (2018).
4. Center for International Earth Science Information Network - CIESIN - Columbia University. Global urban heat island (UHI) data set, 2013. Palisades, NY: NASA Socioeconomic Data and Applications Center. https://doi.org/10.7927/H4H70CRF (2016).
5. Kummu, M., Taka, M. & Guillaume, J. H. A. Gridded global datasets for Gross Domestic Product and Human Development Index over 1990–2015. *Scientific Data*. **5**, 180004 (2018).
6. Center for International Earth Science Information Network - CIESIN - Columbia University. Gridded population of the world, version 4 (GPWv4): Population density, revision 11. Palisades, NY: NASA Socioeconomic Data and Applications Center. https://doi.org/10.7927/H49C6VHW (2018).
7. Huang, C. et al. Mapping the maximum extents of urban green spaces in 1039 cities using dense satellite images. *Environmental Research Letters*. **16**, (2021).
8. Baruch, Z. et al. Increased plant species richness associates with greater soil bacterial diversity in urban green spaces. *Environmental Research*. **196**, (2021).
9. Mills, J. G. et al. Revegetation of urban green space rewilds soil microbiotas with implications for human health and urban design. *Restoration Ecology*. **28**, S322-S334 (2020).
10. Kelepertzis, E. & Argyraki, A. Geochemical associations for evaluating the availability of potentially harmful elements in urban soils: Lessons learnt from Athens, Greece. *Applied Geochemistry*. **59**, 63-73 (2015).
11. Michopoulos, P. et al. Biogeochemistry of lead in an urban forest in Athens, Greece. *Biogeochemistry*. **73**, 345-357 (2005).
12. Schwendenmann, L. & Mitchell, N. D. Carbon accumulation by native trees and soils in an urban park, Auckland. *New Zealand Journal of Ecology*. **38**, 213-220 (2014).
13. Weissert, L. F., Salmond, J. A. & Schwendenmann, L. Variability of soil organic carbon stocks and soil CO2 efflux across urban land use and soil cover types. *Geoderma*. **271**, 80-90 (2016).
14. Madrid, L. et al. Variability in concentrations of potentially toxic elements in urban parks from six European cities. *Journal of Environmental Monitoring*. **8**, 1158-1165 (2006).
15. Sonti, N. F., Hallett, R. A., Griffin, K. L. & Sullivan, J. H. White oak and red maple tree ring analysis reveals enhanced productivity in urban forest patches. *Forest Ecology and Management*. **453**, (2019).

16. Kotze, D. J. et al. Urbanization minimizes the effects of plant traits on soil provisioned ecosystem services across climatic regions. *Global Change Biology*. **27**, 4139-4153 (2021).
17. Pouyat, R. V., Yesilonis, I. D., Russell-Anelli, J. & Neerchal, N. K. Soil chemical and physical properties that differentiate urban land-use and cover types. *Soil Science Society of America Journal*. **71**, 1010-1019 (2007).
18. Pouyat, R. V. et al. A global comparison of surface soil characteristics across five cities: A test of the urban ecosystem convergence hypothesis. *Soil Science*. **180**, 136-145 (2015).
19. Su, Y., Cui, B., Ouyang, Z. & Wang, X. Urban-rural gradients in soil nutrients beneath Chinese pine (Pinus tabulaeformis Carr.) are affected by land-use. *Urban Ecosystems*. **25**, 955-966 (2022).
20. Xu, X. et al. Effects of urban forest types and traits on soil organic carbon stock in Beijing. *Forests*. **12**, (2021).
21. Li, J., Dou, X., Hu, Y., Gan, D. & Li, F. Variation in soil organic carbon mineralization under various land cover types in urban areas. *Acta Ecologica Sinica*. **38**, 112-121 (2018).
22. Luo, S., Mao, Q. & Ma, K. Comparison on soil carbon stocks between urban and suburban topsoil in Beijing, China. *Chinese Geographical Science*. **24**, 551-561 (2014).
23. Zhao, D. et al. The influence of different types of urban land use on soil microbial biomass and functional diversity in Beijing, China. *Soil Use and Management*. **29**, 230-239 (2013).
24. Pavlovic, P. et al. Fractionation of potentially toxic elements (PTEs) in urban soils from Salzburg, Thessaloniki and Belgrade: An insight into source identification and human health risk assessment. *International Journal of Environmental Research And Public Health*. **18**, (2021).
25. Marija, P. et al. Evaluation of urban contamination with trace elements in city parks in Serbia using pine (Pinus nigra Arnold) needles, bark and urban topsoil. *International Journal of Environmental Research*. **11**, 625-639 (2017).
26. Richter, S., Haase, D., Thestorf, K. & Makki, M. Carbon pools of Berlin, Germany: organic carbon in soils and aboveground in trees. *Urban Forestry & Urban Greening*. **54**, (2020).
27. Schmitt-Harsh, M., Mincey, S. K., Patterson, M., Fischer, B. C. & Evans, T. P. Private residential urban forest structure and carbon storage in a moderate-sized urban area in the Midwest, United States. *Urban Forestry & Urban Greening*. **12**, 454-463 (2013).
28. Lompo, D. J.-P. et al. Gaseous emissions of nitrogen and carbon from urban vegetable gardens in Bobo-Dioulasso, Burkina Faso. *Journal of Plant Nutrition and Soil Science*. **175**, 846-853 (2012).
29. Rao, P., Hutyra, L. R., Raciti, S. M. & Finzi, A. C. Field and remotely sensed measures of soil and vegetation carbon and nitrogen across an urbanization gradient in the Boston metropolitan area. *Urban Ecosystems*. **16**, 593-616 (2013).
30. Raciti, S. M., Hutyra, L. R., Rao, P. & Finzi, A. C. Inconsistent definitions of "urban" result in different conclusions about the size of urban carbon and nitrogen stocks. *Ecological Applications*. **22**, 1015-1035 (2012).
31. Enescu, R. E., Dinca, L., Zup, M., Davidescu, S. & Vasile, D. Assessment of soil physical and chemical properties among urban and peri-urban forests: A case study from metropolitan area of Brasov. *Forests*. **13**, (2022).

32. Gonzalez, M., Gomez, E., Comese, R., Quesada, M. & Conti, M. Influence of organic amendments on soil quality potential indicators in an urban horticultural system. *Bioresource Technology*. **101**, 8897-8901 (2010).
33. Bulbovas, P., Camargo, C. Z. S., Ferreira, M. L. & Domingos, M. Anthropic interferences in the nutritional status of tree species growing in urban and peri-urban Atlantic forest remnants. *Urban Forestry & Urban Greening*. **50**, (2020).
34. Ungaro, F. et al. Assessment of joint soil ecosystem services supply in urban green spaces: A case study in Northern Italy. *Urban Forestry & Urban Greening*. **67**, (2022).
35. Zhou, W. et al. Soil fertility evaluation for urban forests and green spaces in Changchun city. *Acta Ecologica Sinica*. **37**, 1211-1220 (2017).
36. Gao, S. et al. Characteristics of soil physicochemical property and its carbon storage in urban forest plantation of Changsha city. *Journal of Central South University of Forestry & Technology*. **30**, 16-22 (2010).
37. Alamgir, M., Islam, M., Hossain, N., Kibria, M. & Rahman, M. Assessment of heavy metal contamination in urban soils of Chittagong city, Bangladesh. *International Journal of Plant & Soil Science*. **7**, 362-372 (2015).
38. Liu, X. et al. The role of land use, construction and road on terrestrial carbon stocks in a newly urbanized area of western Chengdu, China. *Landscape and Urban Planning*. **147**, 88-95 (2016).
39. An, J. et al. Spatial distribution of soil carbon and nitrogen in green lands along an urbanization gradient. *Chinese Journal of Ecology*. **38**, 2780-2787 (2019).
40. Gao, X. et al. Temperature induces soil organic carbon mineralization in urban park green spaces, Chengdu, southwestern China: Effects of planting years and vegetation types. *Urban Forestry & Urban Greening*. **54**, (2020).
41. Wang, H. et al. Soil bacterial diversity is associated with human population density in urban greenspaces. *Environmental Science & Technology*. **52**, 5115-5124 (2018).
42. Feng, H., Zhang, S., Fu, W. & Wang, J. Effects of the age of urban greenbelts on soil physical and chemical properties: A case study of Beibei district of Chongqing. *Journal of Southwest China Normal University. Natural Science Edition*. **37**, 96-104 (2012).
43. Lorenz, K. & Lal, R. in: *Carbon Sequestration in Urban Ecosystems* (eds 139-158 (Springer, 2012).
44. Pradhan, R. et al. Biomass carbon and soil nutrient status in urban green sites at foothills of eastern Himalayas: Implication for carbon management. *Current Research in Environmental Sustainability*. **4**, 100168 (2022).
45. Buil, P. A., Renison, D. & Becerra, A. G. Soil infectivity and arbuscular mycorrhizal fungi communities in four urban green sites in central Argentina. *Urban Forestry & Urban Greening*. **64**, (2021).
46. Raciti, S. M., Groffman, P. M. & Fahey, T. J. Nitrogen retention in urban lawns and forests. *Ecological Applications*. **18**, 1615-1626 (2008).
47. Tan, X., Kan, L., Su, Z., Liu, X. & Zhang, L. The composition and diversity of soil bacterial and fungal communities along an urban-to-rural gradient in south China. *Forests*. **10**, (2019).

48. Nagy, R. C., Lockaby, B. G., Zipperer, W. C. & Marzen, L. J. A comparison of carbon and nitrogen stocks among land uses/covers in coastal Florida. *Urban Ecosystems*. **17**, 255-276 (2014).
49. Li, J., Wang, J., Zeng, W., Guo, L. & Li, X. Vertical distribution characteristics and stock of soil organic carbon in different types of urban green space. *Journal of Anhui Agricultural Sciences*. **39**, 12787-12788+12802 (2011).
50. He, J. et al. Stoichiometric characteristics of soil C,N and P in subtropical forests along an urban-to suburb gradient. *Chinese Journal of Ecology*. **35**, 591-596 (2016).
51. Chen, H. et al. Changes in soil carbon sequestration in Pinus massoniana forests along an urban-to-rural gradient of southern China. *Biogeosciences*. **10**, 6609-6616 (2013).
52. Lin, Y., Xu, Q., Xiao, Q., Bisong, D. & Chen, X. Characteristics of soil organic carbon fractions in the evergreen broad-leaved forests in Guangzhou, China *Ecological Science*. **41**, 114-120 (2022).
53. Chen, X. Soil fertility quality assessment for urban green space in Hangzhou city. *Guizhou Agricultural Sciences*. **40**, 148-150 (2012).
54. Xie, G., Zhang, M., Wu, C. & Wang, J. Study on physical and chemical properties of greenbelt surface soil in Hangzhou city. *Acta Agriculturae Jiangxi*. **25**, 57-61 (2013).
55. Xu, H. et al. Variation characteristics of soil organic carbon composition and management index of Quercus acutissima forests in urban and suburban areas. *Jiangsu Journal of Agricultural Sciences*. **38**, 369-376 (2022).
56. Lu, C., Kotze, D. J. & Setala, H. M. Evergreen trees stimulate carbon accumulation in urban soils via high root production and slow litter decomposition. *Science of the Total Environment*. **774**, (2021).
57. Kargar, M., Jutras, P., Clark, O. G., Hendershot, W. H. & Prasher, S. O. Macro-nutrient availability in surface soil of urban tree pits influenced by land use, soil age, and soil organic matter content. *Urban Ecosystems*. **18**, 921-936 (2015).
58. Huang, M. et al. Responses of soil physical and chemical to differnet functional greenbelts in Hohhot city. *Journal of Inner Mongolia Agricultural University(Natural Science Edition)*. **41**, 10-15 (2020).
59. Gbadegesin, A. & Olabode, M. Soil properties in the metropolitan region of Ibadan, Nigeria: implications for the management of the urban environment of developing countries. *Environmentalist*. **20**, 205-214 (2000).
60. Shen, H. et al. Changes in soil ectomycorrhizal fungi community in Oak forests along the urban-rural gradient. *Forests*. **13**, (2022).
61. An, S. E. & Choonsig, K. Relationships between soil carbon storage and soil properties of urbanparks in Jinju-si, Gyeongsangnam-do. *Korean Journal of Agricultural and Forest Meteorology*. **24**, 115-123 (2022).
62. Beyer, L., Blume, H. P., Elsner, D. C. & Willnow, A. Soil organic matter composition and microbial activity in urban soils. *Science of the Total Environment*. **168**, 267-278 (1995).
63. Nero, B. F., Callo-Concha, D., Anning, A. & Denich, M. Urban green spaces enhance climate change mitigation in cities of the global south: the case of Kumasi, Ghana. In: *Urban Transitions Conference*) (2016).

64. Nero, B. F. & Anning, A. K. Variations in soil characteristics among urban green spaces in Kumasi, Ghana. *Environmental Earth Sciences*. **77**, (2018).
65. Stoma, G., Manucharova, N. & Belokopytova, N. Biological activity of microbial communities in soils of some Russian cities. *Eurasian Soil Science*. **53**, 760-771 (2020).
66. Huang, R. et al. Effect of soil quality of urban greenland on growth conditions of different trees. *Journal of Gansu Agricultural University*. **52**, 113-119 (2017).
67. Edmondson, J. L., Davies, Z. G., McCormack, S. A., Gaston, K. J. & Leake, J. R. Land-cover effects on soil organic carbon stocks in a European city. *Science of the Total Environment*. **472**, 444-453 (2014).
68. Van Geel, M. et al. Variation in ectomycorrhizal fungal communities associated with Silver linden (Tilia tomentosa) within and across urban areas. *Fems Microbiology Ecology*. **94**, (2018).
69. Beesley, L., Moreno-Jimenez, E., Jenn, P. & Lepp, N. W. Carbon and metal(loid)s in parkland and road verge surface soils in the city of Liverpool, UK. *Agronomy-Basel*. **10**, (2020).
70. Trammell, T. L. E., Tripler, C. E., Carper, S. C. & Carreiro, M. M. Potential nitrogen mineralization responses of urban and rural forest soils to elevated temperature in Louisville, KY. *Urban Ecosystems*. **20**, 77-86 (2017).
71. Trammell, T. L. E., Schneid, B. P. & Carreiro, M. M. Forest soils adjacent to urban interstates: Soil physical and chemical properties, heavy metals, disturbance legacies, and relationships with woody vegetation. *Urban Ecosystems*. **14**, 525-552 (2011).
72. Ziter, C. & Turner, M. G. Current and historical land use influence soil-based ecosystem services in an urban landscape. *Ecological Applications*. **28**, 643-654 (2018).
73. Dennis, M., Beesley, L., Hardman, M. & James, P. Ecosystem (Dis)benefits arising from formal and Informal land-use in Manchester (UK); a case study of urban soil characteristics associated with local geen space management. *Agronomy-Basel*. **10**, (2020).
74. Berame, J., ELAZEGUI, E. P., ARENAS, M. C. & OROZCO, J. A. Microclimatic factors and soil characteristics of Arroceros forest park in the city of Manila, Philippines. *Biodiversitas Journal of Biological Diversity*. **22**, (2021).
75. Naylo, A. et al. Trace and major element contents, microbial communities, and enzymatic activities of urban soils of Marrakech city along an anthropization gradient. *Journal of Soils and Sediments*. **19**, 2153-2165 (2019).
76. Beroigui, M. et al. Physicochemical and microbial properties of urban park soils of the cities of Marrakech, Morocco and Torun, Poland: Human health risk assessment of fecal coliforms and trace elements. *Catena*. **194**, (2020).
77. Diaz-Sanz, J., Robert, S. & Keller, C. Parameters influencing run-off on vegetated urban soils: A case study in Marseilles, France. *Geoderma*. **376**, (2020).
78. Han, X.-M. et al. Impacts of reclaimed water irrigation on soil antibiotic resistome in urban parks of Victoria, Australia. *Environmental Pollution*. **211**, 48-57 (2016).
79. Canedoli, C., Ferre, C., Abu El Khair, D., Padoa-Schioppa, E. & Comolli, R. Soil organic carbon stock in different urban land uses: high stock evidence in urban parks. *Urban Ecosystems*. **23**, 159-171 (2020).

80. Vasenev, V. I., Stoorvogel, J. J. & Vasenev, I. I. Urban soil organic carbon and its spatial heterogeneity in comparison with natural and agricultural areas in the Moscow region. *Catena*. **107**, 96-102 (2013).
81. Ivashchenko, K. et al. Microbial C-availability and organic matter decomposition in urban soils of megapolis depend on functional zoning. *Soil & Environment*. **38**, 31-41 (2019).
82. Scharenbroch, B. C., Lloyd, J. E. & Johnson-Maynard, J. L. Distinguishing urban soils with physical, chemical, and biological properties. *Pedobiologia*. **49**, 283-296 (2005).
83. Korneykova, M. V. et al. Urbanization affects soil microbiome profile distribution in the Russian arctic region. *International Journal of Environmental Research And Public Health*. **18**, (2021).
84. Jin, T. et al. Effects of urbanization intensity on glomalin-related soil protein in Nanchang, China: Influencing factors and implications for greenspace soil improvement. *Journal of Environmental Management*. **318**, (2022).
85. Wan, S., Chen, F., Hu, X., Zhang, Y. & Fang, X. Urbanization aggravates imbalances in the active C, N and P pools of terrestrial ecosystems. *Global Ecology and Conservation*. **21**, (2020).
86. Shan, Q., Yu, Y., Yu, J. & Zhang, J. Soil enzyme activities and their indication for fertility of urban forest soil. *Frontiers of Environmental Science & Engineering in China*. **2**, 218-223 (2008).
87. Li, X. et al. Distribution of soil physicochemical properties and microbial biomass in three green lands with different use types in the urban area. *Soils*. **53**, 874-880 (2021).
88. Yang, J., Yu, Y., Chen, Y., Wang, X. & Vasenev, V. I. Content and distribution of organic carbon in urban forest soil in different function areas in Nanjing City, China. *Journal of Nanjing Forestry University. Natural Sciences Edition*. **40**, 22-26 (2016).
89. Zhao, Y.-G., Zhang, G.-L., Zepp, H. & Yang, J.-L. Establishing a spatial grouping base for surface soil properties along urban-rural gradient - A case study in Nanjing, China. *Catena*. **69**, 74-81 (2007).
90. Joyner, J. L. et al. Green infrastructure design influences communities of urban soil bacteria. *Frontiers in Microbiology*. **10**, (2019).
91. Ward, E. B. et al. Positive long-term impacts of restoration on soils in an experimental urban forest. *Ecological Applications*. **31**, (2021).
92. Smith, J., Hallett, R. A., Deeb, M. & Groffman, P. M. Fine-scale soil heterogeneity at an urban site: implications for forest restoration. *Restoration Ecology*. **29**, (2021).
93. Downey, A. E. et al. Soil carbon sequestration in urban afforestation sites in New York City. *Urban Forestry & Urban Greening*. **65**, (2021).
94. Oldfield, E. E. et al. Positive effects of afforestation efforts on the health of urban soils. *Forest Ecology and Management*. **313**, 266-273 (2014).
95. Rosier, C. L., Polson, S. W., D'Amico, V., III, Kan, J. & Trammell, T. L. E. Urbanization pressures alter tree rhizosphere microbiomes. *Scientific Reports*. **11**, (2021).
96. Tang, J. et al. Urban green infrastructure features influence the type and chemical composition of soil dissolved organic matter. *Science of the Total Environment*. **764**, (2021).

97. Foti, L. et al. Topsoil characteristics of forests and lawns along an urban-rural gradient in the Paris region (France). *Soil Use and Management*. **37**, 749-761 (2021).
98. Foti, L. et al. Trace element concentrations along a gradient of urban pressure in forest and lawn soils of the Paris region (France). *Science of the Total Environment*. **598**, 938-948 (2017).
99. Trammell, T. L. E., Pouyat, R. V. & D'Amico, V., III. Heterogeneity in soil chemistry relates to urbanization while soil homogeneity relates to plant invasion in small temperate deciduous forests. *Landscape Ecology*. **37**, 1417-1429 (2022).
100. Zhu, W., Hope, D., Gries, C. & Grimm, N. B. Soil characteristics and the accumulation of inorganic nitrogen in an arid urban ecosystem. *Ecosystems*. **9**, 711-724 (2006).
101. Koerner, B. A. & Klopatek, J. M. Carbon fluxes and nitrogen availability along an urban–rural gradient in a desert landscape. *Urban Ecosystems*. **13**, 1-21 (2010).
102. Guillen-Cruz, G., Rodriguez-Sanchez, A. L., Fernandez-Luqueno, F. & Flores-Renteria, D. Influence of vegetation type on the ecosystem services provided by urban green areas in an arid zone of northern Mexico. *Urban Forestry & Urban Greening*. **62**, (2021).
103. da Silva, R. B., Rosa, J. S., Packer, A. P., Bento, C. B. & de Melo Silva, F. A. A soil quality physical-chemical approach 30 years after land-use change from forest to banana plantation. *Environmental Monitoring and Assessment*. **194**, (2022).
104. Cusack, D. F., Lee, J. K., McCleery, T. L. & LeCroy, C. S. Exotic grasses and nitrate enrichment alter soil carbon cycling along an urban-rural tropical forest gradient. *Global Change Biology*. **21**, 4481-4496 (2015).
105. Paradelo, R., Herbon, C. & Barral, M. T. Composition and chemical properties of the soils of the city of Santiago de Compostela, northwestern Spain. *Journal of Environmental Quality*. **50**, 7-21 (2021).
106. Bae, J. & Ryu, Y. Land use and land cover changes explain spatial and temporal variations of the soil organic carbon stocks in a constructed urban park. *Landscape and Urban Planning*. **136**, 57-67 (2015).
107. Madrid, F., Diaz-Barrientos, E. & Madrid, L. Availability and bio-accessibility of metals in the clay fraction of urban soils of Sevilla. *Environmental Pollution*. **156**, 605-610 (2008).
108. Shi, L., Zheng, L., Mei, X., Yu, L. & Jia, Z. Characteristics of soil organic carbon and total nitrogen under different land use types in Shanghai. *Ying yong sheng tai xue bao = The journal of applied ecology*. **21**, 2279-2287 (2010).
109. Zhang, Q. et al. Soil organic carbon distribution and its relationship with soil physicochemical properties in different forest types of Shanghai City. *Journal of Zhejiang a&f University*. **36**, 1087-1095 (2019).
110. Zhang, Q. et al. Study on changes of soil organic carbon density and influencing factors in urban forest. *Ecologic Science*. **41**, 204-210 (2022).
111. Zhang, W. et al. Diversity patterns and drivers of soil microbial communities in urban and suburban park soils of Shanghai, China. *PeerJ*. **9**, e11231 (2021).
112. Tang, Y., Shi, T., Bu, Y. & Shi, Y. Spatial distribution of soil organic carbon stocks in urban green space with urbanization in Shenyang,China. *Chinese Landscape Architecture*. **35**, 68-73 (2019).

113. Shi, Z. J., Lu, Y., Xu, Z. G. & Fu, S. L. Enzyme activities of urban soils under different land use in the Shenzhen city, China. *Plant Soil and Environment*. **54**, 341-346 (2008).
114. Hu, W. Studies on soil physical-chemical properties and enzymes activities for urban parks and roads green-land in Shenzhen *Master Thesis, Huazhong Agricultural University, Wuhan*. (2008).
115. Stefaner, K. et al. Soil greenhouse gas fluxes from a humid tropical forest and differently managed urban parkland in Singapore. *Science of the Total Environment*. **786**, (2021).
116. Kleine, M. et al. Variation in soil organic carbon stocks in Singapore with forest succession and land management. *Journal of Tropical Ecology*. **38**, 275-284 (2022).
117. Doichinova, V., Zhiyanski, M., Hursthouse, A. & Bech, J. Study on the mobility and bioavailability of PTEs in soils from urban forest parks in Sofia, Bulgaria. *Journal of Geochemical Exploration*. **147**, 222-228 (2014).
118. Rawlins, B. G. et al. Methods for estimating types of soil organic carbon and their application to surveys of UK urban areas. *Soil Use and Management*. **24**, 47-59 (2008).
119. Lorenz, K., Preston, C. M. & Kandeler, E. Soil organic matter in urban soils: Estimation of elemental carbon by thermal oxidation and characterization of organic matter by solid-state C-13 nuclear magnetic resonance (NMR) spectroscopy. *Geoderma*. **130**, 312-323 (2006).
120. Kim, Y. J. & Yoo, G. Suggested key variables for assessment of soil quality in urban roadside tree systems. *Journal of Soils and Sediments*. **21**, 2130-2140 (2021).
121. Horvath, A. et al. A complex soil ecological approach in a sustainable urban environment: soil properties and soil biological quality. *Minerals*. **11**, (2021).
122. Fan, L., Yan, L. & Liu, S. Soil physicochemical property in different urban green lands in Taiyuan. *Journal of Chinese Urban Forestry*. **18**, 30-33 (2020).
123. Zhevelev, H. M., Sarah, P. & Oz, A. The spatial variability and temporal dynamics of soil properties as affected by visitors' pressure in an urban park. *Journal of Environmental Protection*. **4**, 52-64 (2013).
124. Sarah, P. & Zhevelev, H. M. Effect of visitors' pressure on soil and vegetation in several different micro-environments in urban parks in Tel Aviv. *Landscape and Urban Planning*. **83**, 284-293 (2007).
125. Sarah, P., Zhevelev, H. M. & Oz, A. Urban park soil and vegetation: effects of natural and anthropogenic factors. *Pedosphere*. **25**, 392-404 (2015).
126. Matziris, E., Stefanou, S., Papazafeiriou, A. Z., Seilopoulos, D. & Papaioannou, A. Impacts of human activities on soil physical properties of urban green areas: a case study in Thessaloniki city, Greece. *Carpathian Journal of Earth and Environmental Sciences*. **11**, 381-394 (2016).
127. Li, S., Liu, Y. & Cheng, X. Comprehensive evaluation on soil quality in the green land around the central area of Tianjin *Journal of Anhui Agricultural Sciences*. **50**, 61-65+71 (2022).
128. Zhang, C., Li, D., Wei, J., Zhao, L. & Liu, F. Soil respiration and its responses to biotic and abiotic factors in patchy remnant forests and urban green landscapes of Tianjin, China. *Urban Forestry & Urban Greening*. **53**, (2020).

129. Takahashi, T., Amano, Y., Kuchimura, K. & Kobayashi, T. Carbon content of soil in urban parks in Tokyo, Japan. *Landscape and Ecological Engineering*. **4**, 139-142 (2008).
130. Zhang, X., Li, Y. & Wang, P. Analysis of spatial variability of soil organic carbon in Urumqi. *Journal of Arid Land Resources and Environment*. **30**, 117-121 (2016).
131. Rai, P. K., Rai, A. & Singh, S. Change in soil microbial biomass along a rural-urban gradient in Varanasi (UP, India). *Geology, Ecology, and Landscapes*. **2**, 15-21 (2018).
132. Singh, S., Singh, M. K., Kumar, C. M., Soni, P. & Ghoshal, N. Impact of tree plantations on the dynamics of soil aggregates in urban degraded lands in the dry tropics. *Land Degradation & Development*. **32**, 5108-5117 (2021).
133. Upadhyay, S., Singh, R., Verma, P. & Raghubanshi, A. S. Spatio-temporal variability in soil CO2 efflux and regulatory physicochemical parameters from the tropical urban natural and anthropogenic land use classes. *Journal of Environmental Management*. **295**, (2021).
134. Oktaba, L., Odrobinska, D. & Uzarowicz, L. The impact of different land uses in urban area on humus quality. *Journal of Soils and Sediments*. **18**, 2823-2832 (2018).
135. Oktaba, L., Paziewski, K., Kwasowski, W. & Kondras, M. The effect of urbanization on soil properties and soil organic carbon accumulation in topsoil of Pruszków—a medium-sized city in the Warsaw Metropolitan Area, Poland. *Soil Science Annual*. **65**, 10-17 (2014).
136. Wu, H. et al. Soil carbon pool and physic-chemical properties in urban forest of Wuhu. *Chinese Journal of Soil Science*. **49**, 1015-1023 (2018).
137. Zhang, P. et al. Spatial variation and distribution of soil organic carbon in an urban ecosystem from high-density sampling. *Catena*. **204**, (2021).
138. Zhang, X. et al. Effects of vegetation, terrain and soil layer depth on eight soil chemical properties and soil fertility based on hybrid methods at urban forest scale in a typical loess hilly region of China. *Plos One*. **13**, (2018).
139. Fu, Q. et al. Soil microbial distribution depends on different types of landscape vegetation in temperate urban forest ecosystems. *Frontiers in Ecology and Evolution*. **10**, (2022).
140. Tresch, S. et al. Urban soil quality assessment-A comprehensive case study dataset of urban garden soils. *Frontiers in Environmental Science*. **6**, (2018).
141. Beesley, L. Carbon storage and fluxes in existing and newly created urban soils. *Journal of Environmental Management*. **104**, 158-165 (2012).
142. Wavrek, M. T., Jean-Philippe, S. & McKinney, M. L. Ecological and soil data applied to conservation management of an urban forest. *Forests*. **14**, 487 (2023).
143. Adelana, A. O., Aiyelari, E. A., Oluwatosin, G. A. & Are, K. S. Soil properties that differentiate urban land use types with different surface geology in Southwest Nigeria. *Urban Ecosystems*. **26**, 277-290 (2022).
144. Zhang, Z. Y., Yang, X. D., Simay, Z. & Mohammed, A. Health risk evaluation of heavy metals in green land soils from urban parks in Urumqi, northwest China. *Environmental Science And Pollution Research*. **25**, 4459-4473 (2018).
145. Khan, S., Munir, S., Sajjad, M. & Li, G. Urban park soil contamination by potentially harmful elements and human health risk in Peshawar City, Khyber Pakhtunkhwa, Pakistan. *Journal of Geochemical Exploration*. **165**, 102-110 (2016).

146. Serrani, D. et al. Heavy metal load and effects on biochemical properties in urban soils of a medium-sized city, Ancona, Italy. *Environmental Geochemistry and Health*. **44**, 3425-3449 (2022).
147. Du, J., Yu, M., Cong, Y., Lv, H. & Yuan, Z. Soil organic carbon storage in urban green space and its influencing factors: A case study of the 0–20 cm soil layer in Guangzhou city. *Land*. **11**, 1484 (2022).
148. Boim, A. G. F., Patinha, C., Wragg, J., Cave, M. & Alleoni, L. R. F. Respiratory bioaccessibility and solid phase partitioning of potentially harmful elements in urban environmental matrices. *Science of the Total Environment*. **765**, 142791 (2021).
149. Cusack, D. F. Soil nitrogen levels are linked to decomposition enzyme activities along an urban-remote tropical forest gradient. *Soil Biology and Biochemistry*. **57**, 192-203 (2013).
150. Francos, M., Ubeda, X. & Pereira, P. Impact of bonfires on soil properties in an urban park in Vilnius (Lithuania). *Environmental Research*. **181**, 108895 (2020).
151. Rodrigues, S. et al. The influence of anthropogenic and natural geochemical factors on urban soil quality variability: a comparison between Glasgow, UK and Aveiro, Portugal. *Environmental Chemistry Letters*. **7**, 141-148 (2008).
152. Poeplau, C., Marstorp, H., Thored, K. & Kätterer, T. Effect of grassland cutting frequency on soil carbon storage- a case study on public lawns in three Swedish cities. *Soil*. **2**, 175-184 (2016).
153. Gomez-Brandon, M. et al. Influence of land use on the microbiological properties of urban soils. *Applied Soil Ecology*. **175**, (2022).
154. Schifman, L. A., Prues, A., Gilkey, K. & Shuster, W. D. Realizing the opportunities of black carbon in urban soils: Implications for water quality management with green infrastructure. *Science of the Total Environment*. **644**, 1027-1035 (2018).
155. Teng, Y. et al. Environmental vanadium distribution, mobility and bioaccumulation in different land-use Districts in Panzhihua Region, SW China. *Environmental Monitoring and Assessment*. **176**, 605-620 (2011).
156. Mollashahi, H., Szymura, M., Perera, P. C. D. & Szymura, T. H. The effect of grassland type and proximity to the city center on urban soil and vegetation coverage. *Environmental Monitoring and Assessment*. **195**, 599 (2023).
157. Papa, S., Bartoli, G., Pellegrino, A. & Fioretto, A. Microbial activities and trace element contents in an urban soil. *Environmental Monitoring and Assessment*. **165**, 193-203 (2010).
158. Li, L., Li, S., Ma, X. & Yan, Y. Effects of urban green infrastructure designs on soil bacterial community composition and function. *Ecosystems*. 1-11 (2022).
159. Lopes, M. et al. Soil chemical and physical status in semideciduous Atlantic Forest fragments affected by atmospheric deposition in central-eastern São Paulo State, Brazil. *iForest - Biogeosciences and Forestry*. **8**, 798-808 (2015).
160. Milano, V. et al. Collembolan biodiversity in Mediterranean urban parks: impact of history, urbanization, management and soil characteristics. *Applied Soil Ecology*. **119**, 428-437 (2017).

161. McDermot, C. R., Minocha, R., D'Amico III, V., Long, S. & Trammell, T. L. Red maple (Acer rubrum L.) trees demonstrate acclimation to urban conditions in deciduous forests embedded in cities. *PLoS one*. **15**, e0236313 (2020).

162. Ouédraogo, R. A. et al. Short and long-term impact of urban gardening on soil organic carbon fractions in Lixisols (Burkina Faso). *Geoderma*. **362**, 114110 (2020).

163. Peach, M. E., Ogden, L. A., Mora, E. A. & Friedland, A. J. Building houses and managing lawns could limit yard soil carbon for centuries. *Carbon Balance and Management*. **14**, 1-14 (2019).

164. Pižl, V. & Schlaghamerský, J. The impact of pedestrian activity on soil annelids in urban greens. *European Journal of Soil Biology*. **43**, S68-S71 (2007).

165. Rojas, J. A., Dhar, A. & Naeth, M. A. Urban green spaces restoration using native forbs, site preparation and soil amendments-A case study. *Land*. **11**, 498 (2022).

166. Silva, L. et al. Assessment of fatty acid as a differentiator of usages of urban soils. *Chemosphere*. **81**, 968-975 (2010).

167. Škrbić, B. D., Buljovčić, M. & Antić, I. Comprehensive assessment of heavy elements and evaluation of potential human health risk in the urban environment: a case study from Novi Sad, Serbia. *Environmental Science and Pollution Research*. **29**, 38551-38566 (2022).

168. Smith, R. M., Williamson, J. C., Pataki, D. E., Ehleringer, J. & Dennison, P. Soil carbon and nitrogen accumulation in residential lawns of the Salt Lake Valley, Utah. *Oecologia*. **187**, 1107-1118 (2018).

169. Sapkota, M., Young, J., Coldren, C., Slaughter, L. & Longing, S. Soil physiochemical properties and carbon sequestration of Urban landscapes in Lubbock, TX, USA. *Urban Forestry & Urban Greening*. **56**, 126847 (2020).

170. Toth, Z., Dombos, M. & Hornung, E. Urban soil quality deteriorates even with low heavy metal levels: An arthropod-based multi-indices approach. *Ecological Applications*. **33**, e2848 (2023).

171. Xu, X., Wang, C., Sun, Z., Hao, Z. & Day, S. How do urban forests with different land use histories influence soil organic carbon? *Urban Forestry & Urban Greening*. **83**, 127918 (2023).

172. Yang, L., Li, Y., Peng, K. & Wu, S. Nutrients and heavy metals in urban soils under different green space types in Anji, China. *Catena*. **115**, 39-46 (2014).

173. Liu, W. et al. Metal-driven bacterial community variation in urban and suburban park soils of Shanghai, China. *European Journal of Soil Biology*. **115**, 103475 (2023).

174. van Delden, L., Rowlings, D. W., Scheer, C., De Rosa, D. & Grace, P. R. Effect of urbanization on soil methane and nitrous oxide fluxes in subtropical Australia. *Global Change Biology*. **24**, 5695-5707 (2018).

175. Delgado-Baquerizo, M. et al. Biogenic factors explain soil carbon in paired urban and natural ecosystems worldwide. *Nature Climate Change*. **13**, 450-455 (2023).

176. Zhao, F. et al. Urbanization-land-use interactions predict antibiotic contamination in soil across urban-rural gradients. *Science of the Total Environment*. **867**, 161493 (2023).

177. Wang, Z. et al. Soil quality in urban forests under different understory management practices. *Land Degradation & Development*. **34**, 899-910 (2022).

178. Wang, S., Zhu, Z., Yang, L., Li, H. & Ge, B. Body size and weight of pill bugs (Armadillidium vulgare) vary between urban green space habitats. *Animals*. **13**, 857 (2023).
179. Vento, B., Martínez Carretero, E., Moreno, G. & Duplancic, A. Mineral contents in leaves of Morus alba L. (Moraceae) and Platanus hispanica Miller (Platanaceae) from the urban forests of central-western area of Argentina. *Botany Letters*. **164**, 183-192 (2017).
180. Qiao, Z. et al. Urbanization and greenspace type as determinants of species and functional composition of collembolan communities. *Geoderma*. **428**, 116175 (2022).
181. Rahmonov, O., Srodek, D., Pytel, S., Makieieva, N. & Kupka, T. Relationships between heavy metal concentrations in Greater Celandine (Chelidonium majus L.) tissues and soil in urban parks. *International Journal of Environmental Research and Public Health*. **20**, 3887 (2023).
182. Gao, D. et al. Urbanization imprint on soil bacterial communities in forests and grasslands. *Forests*. **14**, 38 (2022).
183. Kong, L., Shi, Z. & Chu, L. M. Carbon emission and sequestration of urban turfgrass systems in Hong Kong. *Science of the Total Environment*. **473-474**, 132-138 (2014).
184. Yesilonis, I. D., James, B. R., Pouyat, R. V. & Momen, B. Lead forms in urban turfgrass and forest soils as related to organic matter content and pH. *Environmental Monitoring and Assessment*. **146**, 1-17 (2008).
185. Yasin, S. Organic carbon sequestration under selected land use in Padang city, West Sumatra, Indonesia. In: *IOP Conference Series: Earth and Environmental Science*). IOP Publishing (2018).
186. Li, Z. Analysis and evaluation of soil fertility characteristics of urban green space in Tangshan. *Modern Horticulture*. **46**, 56-58 (2023).
187. Si, Z. et al. Fertility quality assessment of urban green space soils in Xuzhou city. *Journal of Nanjing Forestry University (Natural Sciences Edition)*. **37**, 60-64 (2013).
188. Sun, S., Chen, D. & Yin, H. Analysis and evaluation of soil fertility characteristics of green space in Hengshui city. *Modern Rural Science and Technology*. 53-55 (2018).
189. Deng, L., Du, N. & Wang, C. Evaluation of soil fertility quality of green space in Qiqihar city. *Heilongjiang Journal of AnimalScience and Veterinary Medical Science*. 171-175 (2017).
190. Abdalla, S. B., Predotova, M., Gebauer, J. & Buerkert, A. Horizontal nutrient flows and balances in irrigated urban gardens of Khartoum, Sudan. *Nutrient Cycling in Agroecosystems*. **92**, 119-132 (2011).
191. Jian, S., Tsai, C., Hseu, Z. & Chen, Z. Baseline concentrations of toxic elements in metropolitan park soils of Taiwan. *Terre. & Aquatic Environ. Toxicol*. **5**, 1-7 (2011).
192. Crispo, M. et al. Heavy metals and metalloids concentrations across UK urban horticultural soils and the factors influencing their bioavailability to food crops. *Environmental Pollution*. **288**, 117960 (2021).
193. Woszczyk, M., Spychalski, W. & Boluspaeva, L. Trace metal (Cd, Cu, Pb, Zn) fractionation in urban-industrial soils of Ust-Kamenogorsk (Oskemen), Kazakhstan-implications for the assessment of environmental quality. *Environmental Monitoring and Assessment*. **190**, 362 (2018).

194. Christel, A. et al. Urban land uses shape soil microbial abundance and diversity. *Science of the Total Environment*. **883**, 163455 (2023).
195. Vladimirovna Afanasyeva, L. & Ayushievna Ayushina, T. Accumulation of heavy metals and biochemical responses in Siberian larch needles in urban area. *Ecotoxicology*. **28**, 578-588 (2019).
196. Bourotte, C., Forti, M. C., Lucas, Y. & Melfi, A. J. Comparison of Polycyclic Aromatic Hydrocarbon (PAHs) concentrations in urban and natural forest soils in the Atlantic Forest (São Paulo State). *Anais da Academia Brasileira de Ciências*. **81**, (2009).
197. Korneykova, M. et al. Soil microbial community of urban green infrastructures in a polar city. *Urban Ecosystems*. **25**, 1399–1415 (2022).
198. Hiller, E. et al. Trace elements in two particle size fractions of urban soils collected from playgrounds in Bratislava (Slovakia). *Environmental Geochemistry and Health*. **42**, 3925-3947 (2020).
199. Luo, X. S. et al. Incorporating bioaccessibility into human health risk assessments of heavy metals in urban park soils. *Science of the Total Environment*. **424**, 88-96 (2012).
200. Mazhari, S. A., Bajestani, A. R. M., Hatefi, F., Aliabadi, K. & Haghighi, F. Soil geochemistry as a tool for the origin investigation and environmental evaluation of urban parks in Mashhad city, NE of Iran. *Environmental Earth Sciences*. **77**, 492 (2018).
201. Velasco, E., Segovia, E., Choong, A. M. F., Lim, B. K. Y. & Vargas, R. Carbon dioxide dynamics in a residential lawn of a tropical city. *Journal of Environmental Management*. **280**, 111752 (2021).
202. Różański, S. Ł., Castejón, J. M. P. & McGahan, D. G. Child risk assessment of selected metal(loid)s from urban soils using in vitro UBM procedure. *Ecological Indicators*. **127**, 107726 (2021).
203. Moura, M., Moita, G. & Moita Neto, J. Analysis and assessment of heavy metals in urban surface soils of Teresina, Piauí State, Brazil: A study based on multivariate analysis. *Comunicata Scientiae*. **1**, 120-127 (2010).
204. Bekier, J., Jamroz, E., Walenczak-Bekier, K. & Uściła, M. Soil organic matter composition in urban soils: A study of Wrocław agglomeration, SW Poland. *Sustainability*. **15**, 2277 (2023).
205. Ferreira, M. D. S., Fontes, M. P. F., Pacheco, A. A., Ker, J. C. & Lima, H. N. Health risks of potentially toxic trace elements in urban soils of Manaus city, Amazon, Brazil. *Environmental Geochemistry and Health*. **43**, 3407-3427 (2021).
206. Famuyiwa, A. O., Davidson, C. M., Ande, S. & Oyeyiola, A. O. Potentially toxic elements in urban soils from public-Access areas in the rapidly growing megacity of Lagos, Nigeria. *Toxics*. **10**, 154 (2022).
207. Mei, Q. et al. Soil fertility evaluation of green space in the main urban area of Hohhot city. *Journal of Yangtze University (Natural Science Edition)*. **17**, 69-72 (2020).
208. Hu, H., Li, R. & Liu, J. Study on physical and chemical properties of greening soil in Kunming residential area. *Journal of Anhui Agricultural Sciences*. **39**, 2129-2131 (2011).
209. Chai, G. Evaluation on soil quality of Fen river park greenland in Linfen.). Shanxi normal university (2017).

210. Karimizadeh, M. & Payandeh, K. Evaluation of Heavy Metals of Lead, Nickel, Cadmium, Vanadium and Some Chemical Parameters in Surface Soils of the City of Khorramabad. *Iranian Journal of Energy & Environment*. **12**, 131-142 (2021).
211. Sosa, D. et al. Polycyclic aromatic hydrocarbons and polychlorinated biphenyls in urban and semi-urban soils of Havana, Cuba. *Journal of Soils and Sediments*. **19**, 1328-1341 (2018).
212. Parlak, M., Tunçay, T. & Botsou, F. Heavy metals in soil and sand from playgrounds of Çanakkale city (Turkey), and related health risks for children. *Sustainability*. **14**, 1145 (2022).
213. Adhikari, G. & Bhattacharyya, K. G. Correlation of soil organic carbon and nutrients (NPK) to soil mineralogy, texture, aggregation, and land use pattern. *Environmental Monitoring and Assessment*. **187**, 735 (2015).
214. O'Riordan, R., Davies, J., Stevens, C. & Quinton, J. N. The effects of sealing on urban soil carbon and nutrients. *Soil*. **7**, 661-675 (2021).
215. Molla, A., Zhang, W., Zuo, S., Ren, Y. & Han, J. A machine learning and geostatistical hybrid method to improve spatial prediction accuracy of soil potentially toxic elements. *Stochastic Environmental Research and Risk Assessment*. **37**, 681-696 (2022).
216. Saavedra-Romero, L. d. L., Alvarado-Rosales, D., Martínez-Trinidad, T. & Hernández-de la Rosa, P. Physical and chemical properties of the urban soil in the San Juan de Aragon Park, Mexico City. *Revista Terra Latinoamericana*. **38**, 529-540 (2020).
217. Marquez‐Bravo, L. G. et al. Concentrations of polycyclic aromatic hydrocarbons in New York City community garden soils: Potential sources and influential factors. *Environmental Toxicology and Chemistry*. **35**, 357-367 (2016).
218. Johnston, M. R., Balster, N. J. & Zhu, J. Impact of residential prairie gardens on the physical properties of urban soil in Madison, Wisconsin. *Journal of Environmental Quality*. **45**, 45-52 (2016).
219. Chahal, M. K., Toor, G. S. & Brown, P. Trace metals and polycyclic aromatic hydrocarbons in an urbanized area of Florida. *Soil and Sediment Contamination: An International Journal*. **19**, 419-435 (2010).
220. Probst, M., Gómez-Brandón, M., Herbón, C., Barral, M. T. & Paradelo, R. Fungal-bacterial associations in urban allotment garden soils. *Applied Soil Ecology*. **188**, 104896 (2023).
221. Sun, P., Li, W. & Guo, S. Effects of the soil characteristics of four different types of green space on plant root activity. *Heilongjiang Agricultural Science*. 45-50 (2016).
222. Di, D. et al. Soil chemical properties and heavy metal contamination in different green space types of main urban area, Baoding city. *Journal of Northeast Forestry University*. **50**, 88-95+110 (2022).
223. Lv, W., Wang, S., Liu, X. & Rong, L. Influence of rainfall on soil respiration in karst urban green space. *Earth and Environment*. **39**, 174-180 (2011).
224. Sun, L. & Yang, J. Analysis of soil nutrient heterogeneity of urban green space in Maiji District of Tianshui City. *Gansu Science and Technology Vertical and Horizontal*. **50**, 17-19 (2021).

225. Zhang, Z., Li, Y., Wang, W., Xu, M. & Pang, Z. Effects of different litter remaining characteristics of typical vegetation communities in urban green spaces on the soil physicochemical properties. *Journal of Forest and Environment*. **41**, 132-139 (2021).
226. Zhang, X., Shi, Y. & Shi, Y. Physical and chemical properties in green space of Harbin City. *Protection Forest Science and Technology*. 25-27 (2015).
227. Zhou, W. et al. Soil fertility and spatial variability of urban green land in Harbin. *Scientia Silvae Sinicae*. **54**, 9-17 (2018).
228. Amossé, J. et al. Patterns of earthworm, enchytraeid and nematode diversity and community structure in urban soils of different ages. *European Journal of Soil Biology*. **73**, 46-58 (2016).
229. Contosta, A. R., Lerman, S. B., Xiao, J. & Varner, R. K. Biogeochemical and socioeconomic drivers of above- and below-ground carbon stocks in urban residential yards of a small city. *Landscape and Urban Planning*. **196**, 103724 (2020).
230. Gautam, P., Young, J. R., Sapkota, M., Longing, S. & Weindorf, D. C. Soil carbon sequestration in bermudagrass golf course fairways in Lubbock, Texas. *Agronomy Journal*. **112**, 148-157 (2020).
231. Selhorst, A. & Lal, R. Net carbon sequestration potential and emissions in home lawn turfgrasses of the United States. *Environmental management*. **51**, 198-208 (2013).
232. Trammell, T. L. et al. Urban soil carbon and nitrogen converge at a continental scale. *Ecological Monographs*. **90**, e01401 (2020).
233. Livesley, S. J. et al. Soil-atmosphere exchange of carbon dioxide, methane and nitrous oxide in urban garden systems: impact of irrigation, fertiliser and mulch. *Urban Ecosystems*. **13**, 273-293 (2010).
234. Cachada, A. et al. The variability of polychlorinated biphenyls levels in urban soils from five European cities. *Environmental Pollution*. **157**, 511-518 (2009).
235. Biasioli, M. et al. Potentially toxic elements contamination in urban soils: a comparison of three European cities. *Journal of Environmental Quality*. **36**, 70-79 (2007).
236. Wang, Q. et al. Spatial change in glomalin-related soil protein and its relationships with soil enzyme activities and microbial community structures during urbanization in Nanchang, China. *Geoderma*. **434**, 116476 (2023).
237. Fausak, L., Watkinson, A. D., Dy, K. & Lavkulich, L. Assessment of metal contamination in soil and vegetation along the Arbutus Greenway in Vancouver, British Columbia. *Plant and Soil*. **464**, 593-604 (2021).
238. Bartkowiak, A., Lemanowicz, J. & Lamparski, R. Assessment of selected heavy metals and enzyme activity in soils within the zone of influence of various tree species. *Scientific Reports*. **10**, 14077 (2020).
239. Hung, W., Hernandez-Cira, M., Jimenez, K., Elston, I. & Jay, J. A. Preliminary assessment of lead concentrations in topsoil of 100 parks in Los Angeles, California. *Applied Geochemistry*. **99**, 13-21 (2018).
240. Martinez, N. G., Bettez, N. D. & Groffman, P. M. Sources of variation in home lawn soil nitrogen dynamics. *Journal of environmental quality*. **43**, 2146-2151 (2014).

241. Sundarapandian, S. et al. Soil organic carbon stocks in different land uses in Pondicherry university campus, Puducherry, India. *Tropical Plant Research*. **3**, 10-17 (2016).
242. Saha, A. & Handique, S. Variation of soil organic carbon in a university campus in temperate North-eastern India. *Environmental Challenges*. **7**, 100493 (2022).
243. Singh, D. P., Gadi, R. & Mandal, T. K. Levels, sources, and toxic potential of polycyclic aromatic hydrocarbons in urban soil of Delhi, India. *Human and Ecological Risk Assessment: An International Journal*. **18**, 393-411 (2012).
244. Yang, H., Xiong, H., Chen, X., Wang, Y. & Zhang, F. Identifying the influence of urbanization on soil organic matter content and pH from soil magnetic characteristics. *Journal of Arid Land*. **7**, 820-830 (2015).
245. Lacatusu, R., Lacatusu, A.-R., Lungu, M. & Breaban, I. Macro-and microelements abundance in some urban soils from Romania. *Carpathian Journal of Earth and Environmental Sciences*. **3**, 75-83 (2008).
246. Hyun, J., Kim, Y. J., Kim, A., Plante, A. F. & Yoo, G. Ecosystem services-based soil quality index tailored to the metropolitan environment for soil assessment and management. *Science of the Total Environment*. **820**, 153301 (2022).
247. Maisto, G., Milano, V. & Santorufo, L. Relationships among site characteristics, taxonomical structure and functional trait distribution of arthropods in forest, urban and agricultural soils of Southern Italy. *Ecological Research*. **32**, 511-521 (2017).
248. Suleiman, R. & Ibrahim, J. Assessment of Soil Physical and Chemical Properties under Vegetable Cultivation in Abuja Metropolitan Area,Nigeria. *Zaria Geographer*. **24**, 89-99 (2017).
249. Mao, Q. et al. Spatial heterogeneity of urban soils:the case of the Beijing metropolitan region,China. *Ecological Processes*. **3**, 11 (2014).
250. Tobias, S., Davies, M., Imhof, C. S., Psomas, A. & Boivin, P. Greening and browning of urban lawns in Geneva (Switzerland) as influenced by soil properties. *Geoderma Regional*. **34**, e00677 (2023).
251. Wiseman, C. L., Zereini, F. & Püttmann, W. Metal and metalloid accumulation in cultivated urban soils: A medium-term study of trends in Toronto, Canada. *Science of the Total Environment*. **538**, 564-572 (2015).
