# Supplementary material for: Global distribution of surface soil organic carbon in urban greenspaces
Source: Nat Commun. 2024 Jan 27;15:806. doi: 10.1038/s41467-024-44887-y (PMC11258340; doi:10.1038/s41467-024-44887-y)
Supplement: Supplementary file 3 — Description of Additional Supplementary Files [file 41467_2024_44887_MOESM3_ESM.pdf]

Description of Additional Supplementary Files

File Name: Supplementary Data 1
Description: The city-level soil organic carbon density and soil organic carbon stock of urban greenspaces for mid- and large cities (urban population > 0.5 million)

ID: ID of the predicted cities
Lat: Latitude (°)
Lon: Longitude (°)
City: Name of the predicted cities
Country: Country of the predicted cities
Continent: Continent of the predicted cities
USGA: Total urban green space area (km^2)
SOCD: Predicted soil organic carbon density (0-20 cm) (Mg/ha)
SOCS: Predicted soil organic carbon stock (0-20 cm) (Tg)

File Name: Supplementary Data 2
Description: The national-level soil organic carbon density and soil organic carbon stock of urban greenspaces

ID: ID of the predicted cities
Country: Country
UA: Total urban built-up area (km^2)
USGA: Total urban green space area (km^2)
SOCD: Predicted soil organic carbon density (0-20 cm) (Mg/ha)
SOCS: Predicted soil organic carbon stock (0-20 cm) (Tg)

File Name: Supplementary Data 3
Description: The SOC-U dataset

ID: ID of the urban greenspace sites
City: Name of the sampled cities
Country: Country of the sampled cities
Continent: Continent of the sampled cities
Lat: Latitude (°)
Lon: Longitude (°)
Climate: Climate type of the sampled cities
Sampling year: Soil sampling year
Depth: Soil depth (cm)
Landuse: Dominant landuse type of the sampled sites
Dominant vegetation: Dominant vegetation type of the sampled sites
Elevation: Elevation of the sampled cities (m)
MAT: Mean annual temperature (℃)
MAP: Mean annual precipitation (mm)
Temperature seasonality: Temperature seasonality (10-2 ℃)
Precipitation seasonality: Precipitation seasonality (%)
PD: Urban population density (people/km2)

GDPP: Gross domestic product per capita (USD)
UGI: Urban greenness index
UHI: Urban heat island index (℃)
pH: Soil pH
Clay: Soil clay fraction (%)
TN: Soil total nitrogen concentration (g/kg)
TP: Soil total phosphorus concentration (g/kg)
BD: Soil bulk density (g/cm3)
SOCC: Soil organic carbon concentration (g/kg)
SOCD: Soil organic carbon density (Mg/ha)
SOCD20: Soil organic carbon density (0-20 cm) (Mg/ha)
Reference: Original literature
Site number: Number of the sampled sites
